# E-Prevention: Advanced Support System for Monitoring and Relapse Prevention in Patients with Psychotic Disorders Analyzing Long-Term Multimodal Data from Wearables and Video Captures

**DOI:** 10.3390/s22197544

**Published:** 2022-10-05

**Authors:** Athanasia Zlatintsi, Panagiotis P. Filntisis, Christos Garoufis, Niki Efthymiou, Petros Maragos, Andreas Menychtas, Ilias Maglogiannis, Panayiotis Tsanakas, Thomas Sounapoglou, Emmanouil Kalisperakis, Thomas Karantinos, Marina Lazaridi, Vasiliki Garyfalli, Asimakis Mantas, Leonidas Mantonakis, Nikolaos Smyrnis

**Affiliations:** 1 School of ECE, National Technical University of Athens, 157 73 Athens, Greece; 2 Department of Digital Systems, University of Piraeus, 185 34 Pireas, Greece; 3 BLOCKACHAIN PC, 555 35 Thessaloniki, Greece; 4 Laboratory of Cognitive Neuroscience and Sensorimotor Control, University Mental Health, Neurosciences and Precision Medicine Research Institute “COSTAS STEFANIS”, 115 27 Athens, Greece; 5 1st Department of Psychiatry, Eginition Hospital, Medical School, National and Kapodistrian University of Athens, 115 28 Athens, Greece; 6 2nd Department of Psychiatry, University General Hospital “ATTIKON”, Medical School, National and Kapodistrian University of Athens, 124 62 Athens, Greece

**Keywords:** anomaly detection, autoencoder architectures, biometric indexes, deep learning, digital phenotyping, facial expressions, psychotic disorders, relapse detection, spontaneous speech, wearable technologies

## Abstract

Wearable technologies and digital phenotyping foster unique opportunities for designing novel intelligent electronic services that can address various well-being issues in patients with mental disorders (i.e., schizophrenia and bipolar disorder), thus having the potential to revolutionize psychiatry and its clinical practice. In this paper, we present e-Prevention, an innovative integrated system for medical support that facilitates effective monitoring and relapse prevention in patients with mental disorders. The technologies offered through e-Prevention include: (i) long-term continuous recording of biometric and behavioral indices through a smartwatch; (ii) video recordings of patients while being interviewed by a clinician, using a tablet; (iii) automatic and systematic storage of these data in a dedicated Cloud server and; (iv) the ability of relapse detection and prediction. This paper focuses on the description of the e-Prevention system and the methodologies developed for the identification of feature representations that correlate with and can predict psychopathology and relapses in patients with mental disorders. Specifically, we tackle the problem of relapse detection and prediction using Machine and Deep Learning techniques on all collected data. The results are promising, indicating that such predictions could be made and leading eventually to the prediction of psychopathology and the prevention of relapses.

## 1. Introduction

Digital phenotyping [1] is a nascent exciting interdisciplinary field motivated by the broad adoption of wearable products (i.e., smartwatches and fitness trackers) in our daily lives. The term encompasses the quantification of human behavior and traits (the “phenotype’’) in situ by utilizing the sensors included in these devices. Such wearables collect multimodal data, usually using accelerometers, gyroscopes and heart rate monitors among others, to measure the user’s physical activity and kinetic activity, such as micro-movements and autonomic function [2,3,4].

This abundance of sensory data has kickstarted the development of several applications focused on general user and health monitoring, as well as other predictive analytic tasks, e.g., emotional well-being [5,6,7,8], sleep tracking [9,10], eating [11], agitation [12] and physical activity detection [13,14]. Many works have also focused on identifying behavioral and biometric markers, which can be extracted from such data and provide insights into disciplines such as general medicine [15] or sports [16], examining various well-being problems [17,18,19]. The success of these applications has also increased the interest in psychological health for human wellness. Increasing evidence shows that such markers could be introduced into clinical psychiatry [20] by employing them to examine depression [21,22,23], bipolar disorder [24], or schizophrenia [25]. Especially for people with mental disorders, sensing biometric markers of interest unobtrusively through passive, continuous and long-term monitoring could prove effective in improving wellness and the course of the disorder.

Psychosis is a spectrum of conditions triggered by various etiopathogenic mechanisms that affect the Central Nervous System (CNS), resulting in common symptoms [26]. Over the last 60 years, various studies of such psychiatric conditions have been conducted in neurobiology and neurophysiology; however, their causes still remain unknown. The consequence of this is that no effective biomarkers for either diagnosis or prediction of the course of psychotic symptomatology have yet been discovered; thus, now the utilization of such markers for timely diagnosis and prevention of psychotic relapses constitutes one of the most prominent study areas in psychiatry [27,28,29]. Actually, early identification of worsening symptoms in the early stages of the psychotic process, and early prevention of relapses have been found to contribute significantly to better outcomes of the disorder [30,31,32] and in preventing the catastrophic effects that relapses often have on patients’ lives [33]. Given that psychosis is evolving continuously and relapse is a biological process that develops over time [34,35,36], it would be reasonable to anticipate variations in the behavior of such biomarkers that are related to and probably precede the onset and/or worsening of such mental disorders. Some of the typical early warning symptoms of psychiatric conditions include rigidity, tremor, abrupt arm movements, atypical motions or postures, and withdrawal from outdoor activities, among others [37,38]. So, on the above basis, it would be possible to create an intelligent system that could continuously and passively measure human behavior to detect these changes and prevent psychotic relapses before symptoms are fully expressed.

Computational signal processing is actually the keystone that supports the mapping from raw sensor data to representations of behaviors and mental states (for a review see [39]). The pipeline begins with raw signals from physiological or wearable sensors; afterwards, the signals have to be modeled appropriately to extract meaningful information, whereas, in the final stage, machine or deep learning has to be used to make inferences on mental states to support human or autonomous decision making, which is indeed one of the main contributions of the ***e-Prevention*** project that is described in this work. Apart from that, usually an integrated and intelligent system has to be built from scratch so as to render it as accessible and practical as it can be for non-tech-savvy users (i.e., patients and clinicians); this constitutes the other main contribution of this work.

### Contributions and Overview

The work described in this paper concerns the ***e-Prevention*** (More info can be found in: http://eprevention.gr (accessed on 20 August 2022)) project—a long-term, more than three-year study—aiming to develop innovative and advanced electronic services for medical monitoring and support that will facilitate effective monitoring and relapse prevention in patients with mental disorders (i.e., bipolar disorder and schizophrenia). The project has developed an innovative integrated system, which offers the following technologies: (1) long-term continuous monitoring and recording of biometric and behavioral indices through a non-intrusive and simple wearable sensor, namely a smartwatch; (2) a portable mobile device (tablet) installed in the patient’s house, in order to record short audio-visual videos of the patient while being interviewed by the clinician, with the aim to collect social features, including speech and facial expressions and; (3) automatic and systematic storing of these data in a Cloud server. Through these, the ultimate goal of the e-Prevention project is the identification of markers and feature representations that correlate with and can predict the mood and psychopathology of patients, serving as measures of prediction and thus eventually preventing possible relapses.

In order to accomplish this, we performed a thorough statistical exploration of the differences presented in biomarkers between healthy controls and patients with mental disorders (i.e., schizophrenia and bipolar disorder), identifying significant differences between the groups. This statistical analysis—using well-established and reliable statistical tests, such as the Kruskal–Wallis H test and Dunn’s tests with the respective corrections—is based on an innovative and extensive multisensory and multiscale data analysis of long-term continuous behavioral (i.e., accelerometer and gyroscopic data) and physiological markers (i.e., heart rate and heart rate variability) that were collected passively for a long-term period, which lasted almost one year (∼110,000 h across all users). For this analysis, classical and more advanced linear and nonlinear signal processing techniques are compared with the goal to extract the most appropriate behavioral and physiological descriptors, showing significant statistical differences between the groups.

Based on these representations that showed to be more significant, we continued by examining various Deep Learning techniques so as to detect relapses in patients using physiological data acquired from the smartwatch. Additionally, we explored the usefulness of spontaneous speech data obtained during weekly or bi-weekly interviews conducted between patients and clinicians having the same goal of detecting and predicting relapses. Both tasks were tackled in an anomaly detection framework, and our models were implemented using both personalized and global schemes. Finally, we explored the potential of automatically recognizing alterations in psychopathology, determined through the Positive and Negative Syndrome Scale (PANSS) [40], using facial cues and classical supervised machine learning algorithms. The results that we obtained are promising in all tasks that we handled, i.e., relapse detection and prediction using physiological data, audio data, the fusion of audio with physiological data, and finally, video data for the evaluation of facial cues in patients with mental disorders.

The remainder of this paper is organized as follows: In Section 2, the related work is presented, whereas in Section 3, we describe the overall architecture of the e-Prevention system and the various subsystems that were developed in order to collect and store all involved data. In Section 4, the recruitment protocol of the volunteers, thus controls and patients, is described. In Section 5, we go into more detail regarding the methodologies used in order to carry out the statistical analysis conducted for the identification of differences in physical activity and autonomic function patterns between psychotic patients and controls using wearable data (Section 5.1); additionally, we present our research results on detection and prediction of relapses and general psychopathology using physiological data (Section 5.2) or audio-visual cues obtained from the interviews conducted between patients and clinicians (Section 5.3 and Section 5.4). Finally, in Section 6, we conclude our work.

## 2. Related Work

Physical activity is an everyday routine for all of us, i.e., when walking to work or to the market; however, for people with mental disorders, such activities are usually disrupted during or even before a relapse. Nowadays, there is growing scientific evidence that such activity could constitute the most consistent indicator in order to classify various episode types in such patients [37], something that is usually performed using more traditional monitoring with specialized questionnaires appropriately designed to this end [41]; however, such methods are heavily biased by the “current” state of the patient. Several works [41,42] have also suggested employing today’s technological advancements and digital phenotyping for accurate and continuous patient monitoring to reduce the impact of mental illness offering clinically relevant information for understanding how daily routines affect symptomatology [43], aiming to increase the effectiveness of treatments. So, the current advances in popular and affordable technologies—such as accelerometers among others in wearables and smartphones— open the possibility for the non-intrusive acquisition of activity and physiological data, which could actually both transform hospital-centered healthcare practice into proactive, individualized care and improve the patient’s course of life.

There are works that have tried to tackle this problem offering promising evidence for using such sensorial data. In [37], accelerometer and audio information from smartphones were used to classify the state of five patients with bipolar disorder by collecting information about their real-life activities over a 12-week time period, showing that the course of mood episodes or relapses can be predicted with high confidence. In [44], statistical methods were employed to characterize accelerometer-derived activity patterns (collected over a week) from 99 adults with heterogeneous mental illnesses, claiming that activity patterns varied between different disorders, whereas the rich nature of human movement captured by accelerometry during wake and sleep could be quantified. In [45], simple features were extracted from linear accelerations and angular velocities from patients with Parkinsonian tremors (a usual symptom of patients with psychiatric disorders), concluding that both linear acceleration and rotational motion of the wrist improve the prediction accuracy for detecting tremors. Finally, in [46], changes in mobility and social behavior, measured through smartphones, could identify statistically significant anomalies in patient behavior during the days prior to a relapse.

Previous works have mostly used smartphones [47] and focused mainly on social features such as text messages, call duration, and sleep duration among others [46,48,49] lasting from some hours to a few weeks [46,50,51], with some exceptions [48]. Compared to smartphones, wearable sensors are unobtrusive, lightweight and can be used for the monitoring of daily activities [52]. It has also been shown that people with mental disorders are comfortable and willing to integrate them into their daily life, something that supports the fact that by using smartwatches we could go beyond feasibility and underscore the novel physiological and activity data that can be easily collected with low cost [53].

Supervised learning approaches to correlating the appearance of relapses with physiological data have mostly focused on either statistical significance testing [54] or classification of hand-crafted features using traditional machine learning algorithms [51]. Consequently, a variety of feature representations have been proposed in such medical settings using data from wearables; many of those are related to Heart Rate Variability (HRV), which describes the variation in heartbeat intervals and is considered a reliable quantitative measure of Autonomic Nervous System (ANS) activity, and have been reported in a wide variety of psychiatric disorders [54,55]. Some of the most frequently used linear methods for HRV analysis are based on the mean Heart Rate (HR) [54], the mean HRV [56], the standard deviation (SD), the root mean square of successive differences (RMSSD) [56], and the proportion of consecutive RR intervals that differ by more than 50 ms (pNN50) [57], as well as various spectral features, specifically, the LF and HF, and thus, the power in a low- or high-frequency band, along with the ratio of LF-to-HF [56,57]. However, over the past decade, there has been an increasing emphasis on applying nonlinear methods to characterize cardiac function; fluctuations in heart rate have been reported to present irregular variability that suggests nonlinear behavior [58]. Such methods include detrended fluctuation analysis [59], sample entropy [60], Poincare plots [61], and fractal dimension [62,63,64] among others. There is evidence that nonlinear metrics are superior predictors of cardiac and autonomic dysfunction [65,66,67] when compared to more traditional time and frequency domain analyses; however, they have not yet been extensively examined in research areas that include people with mental disorders. In [56,68], a review of linear and nonlinear methods can be found, indicating the most significant; whereas commonly used HRV measures and their diagnostic uses can be found in [57].

Apart from signals collected from wearables, a plethora of other modalities, such as speech, facial cues and social activity data have been shown to contain information related to such biomarkers that could correlate with the appearance of relapses. Specifically, speech has been shown to be indicative of both the emotional state of a person [69] and relapsing mental conditions [70]. For instance, bipolar disorder can be characterized by longer pauses in between utterances of the patients and increased pitch and formant frequencies, whereas relapsing schizophrenic patients show decreased pitch and formant frequencies, coupled with a lower speech rate and longer pauses in between utterances [70]. Supervised approaches towards the detection of relapsing mental conditions have utilized both hand-crafted features [71,72,73] and deep learning [74,75]. In the first case, features are extracted either from short-time frames of speech signals [71] or whole interviews [72,73], whereas deep learning algorithms have been applied in low-level descriptors of raw speech signals [74] or spectrograms [75].

A promising area that has lately drawn interest from both the computer vision and machine learning communities is the automatic recognition of depression using facial cues and facial expressions. For example, in [76], the viability of automatic depression detection was demonstrated by comparing medical assessments with automatically measured facial actions using the Active Appearance Model (AAM), which was also used in [77], in order to extract eye movement features from videos to classify subjects as depressed or not. Deep learning algorithms that train discriminant feature representations have also been utilized in aided clinical diagnosis [78], obtaining state-of-the-art results, even though the use of hand-crafted features has been common practice for years in the specific task. In the case of deep learning operating on videos, it is usual to exploit spatial and temporal information independently (e.g., by cascading a 2D CNN and then a recurrent NN), while deteriorating, however, the modeling of spatio-temporal relationships [79,80]. A deep two-stream architecture has also been used to exploit facial appearance and optical flow [81], while during recent years, 3D CNNs, such as the C3D network [82,83], have also been proposed to utilize spatio-temporal correlations increasing the detection accuracy.

Apart from methods based on supervised learning, another approach that could be used for relapse detection is sensor-based anomaly detection, the importance of which has been highlighted during recent years and the pandemic, through the clinical mass adoption of telehealth [84]. Anomaly detection, motivated by the rarity of appearance of abnormal (anomalous) events, as well as the potential lack of strong labels, concerns the development of either weakly-supervised or unsupervised anomaly detection algorithms [85]. This is especially significant in mental health monitoring, where the availability of data corresponding to relapsing states is scarce. Such unsupervised anomaly detection algorithms, such as, for instance, autoencoder neural networks, have been developed and applied on audio signals [86,87] and medical images [88], as well as data collected from various passive sensors [48,89,90,91].

## 3. The E-Prevention System

### 3.1. Overview

The innovative methodology of the e-Prevention approach introduces several operational challenges for the overall system and for the specific processes, which are realized by the different system components. These challenges are related mainly to the ultimate objective of the project, namely to create an online framework for the effective communication of different user entities and the automated collection, management and analysis of multimodal data (biomarkers) related to psychotic disorders. This complex environment requires the design and implementation of a robust and modular platform and user-friendly applications able to address the project objectives, provide the necessary levels of usability and automation for the non-tech-savvy users that access it (e.g., patients and clinicians), and at the same time incorporate state-of-the-art technologies for data analysis, signal processing and machine/deep learning to support the scientific requirements of the researchers and analysts involved.

**Figure 1:**
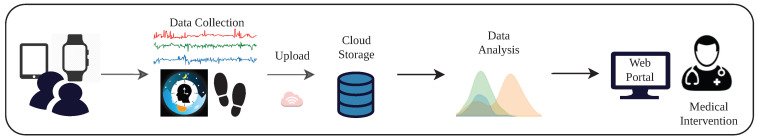
The e-Prevention system’s paradigm.

Figure 1 presents the architecture of the integrated e-Prevention system showing the users’ interactions with its various subsystems. There are four main subsystems:

(1) **Data collection**: which is responsible for collecting the data required by the e-Prevention project consisting of the following elements: (i) The *smartwatch* and its application, which is responsible for collecting biomarkers, such as kinetic data through the accelerometer and gyroscope sensors and heart rate variability (HR with the corresponding RR-intervals) using a PPG-based (Photoplethysmography) non-invasive heart rate monitor on a daily basis (24/7) from patients. (ii) The *tablet* and its application, which is responsible for recording the weekly or biweekly interviews between clinicians and patients. Both dedicated applications were developed for the purposes of the project;
(2) **Cloud storage**: which is responsible for obtaining and maintaining the data collected in the data collection subsystem. The information entered by the clinicians in the web portal, described next, is also stored in the cloud storage;
(3) **Data analysis**: where the data are processed and all results and conclusions are extracted;
(4) **Web Portal**: consisting of the *dashboard* allowing the clinicians to access and process the patients’ personal information. It also provides the ability to present visualizations and statistics as they emerge after data analysis. Finally, it also incorporates the *video interview mechanism*, which constitutes the interface for the interviews conducted between clinicians and patients.

### 3.2. Cloud Architecture

**Figure 2:**
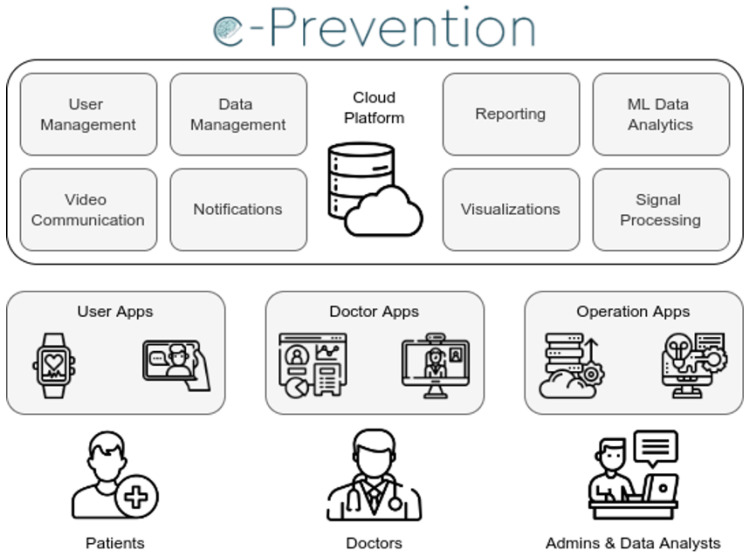
The overall architecture of the proposed system.

The overall system architecture includes a cloud-based platform with several active components and client applications for the different users involved in the project (Figure 2). The cloud platform coordinates the various operations of the system and focuses on the efficiency of the user and data management processes. Additionally, all data produced are hosted on this cloud platform, which is on the premises of the National Research Network and Technology (GRNET—https://grnet.gr (accessed on 20 August 2022)). GRNET provides cloud computing services in the form of Infrastructure as a Service, called “∼okeanos”, through which any user of the academic and research community can create multilevel virtual infrastructures, having the possibility to either use them as virtual disks or virtual storage in the cloud.

The user management component maintains the anonymized list of all participants (controls and patients) in the project and is responsible for the clinicians’ and the involved researchers’ authentication and authorization. This component realizes role-based access control, allowing only specific user entities to interact with data and system services. Furthermore, this component includes device management capabilities associating sensors and devices to specific users and simplifying the processes for user onboarding, sign in and monitoring.

Data management is also at the core of the e-Prevention cloud platform with the aggregation of multimodal data from different sources and their persistence in the databases and object storage of the platform. More specifically, this component provides the public endpoints for the synchronization of data with the client applications and for their processing and analysis from the respective tools of the platform. In addition, the platform provides real-time video communication functionality between patients and clinicians, and also notifications for important events related to the management of the devices (i.e., smartwatch and tablet) or the health and mental condition of patients following the analysis of the ingested data.

Finally, the platform also includes several tools for analysis of the data that exploit the various cloud resources, which are available and provide results in both online (as the data are ingested into the system) and offline modes by exploiting state-of-the-art signal processing and machine learning frameworks and methodologies. These tools are used by both clinicians and data scientists of the project creating visualizations and periodic reports for the patients that are monitored through the platform.

### 3.3. Smartwatch Application

In order to select the appropriate smartwatch device that best accommodated the requirements of the e-Prevention project (i.e., collection and transmission of large amounts of raw biosignals) we experimented with a number of different commercially available devices. After careful consideration, we selected the Samsung Gear S3 Frontier smartwatch, which has the ability to store and transmit data from its accelerometer, gyroscope and PPG (photoplethysmography) sensors. Note that the PPG sensor provides not only the heart rate but also the heartbeat interval period value (R-R interval). The storage capacity of the device is 4 GB and its WiFi interface allows for wireless connection and transmission of data, thus removing the need for a smartphone for connectivity.

**Figure 3:**
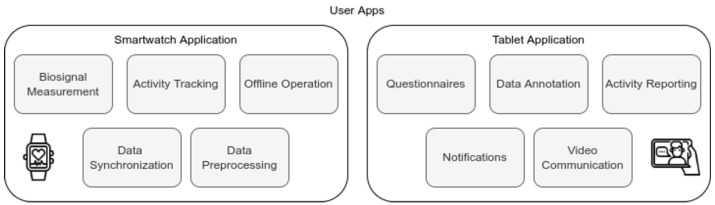
Smartwatch and tablet application functionalities.

In order to use the capabilities of the smartwatch, we built an in-house application using the Tizen Studio environment [92]. The application includes several components for offline operation, local data management, and parallel preprocessing of data, for efficient synchronization without data loss. For more information see also [93]. More specifically, the developed application includes the following modules: (a) the data collection module, which is capable of collecting a wide range of the user’s biosignals (see Table 1) by exploiting the device’s sensors providing at the same time the required levels of automation and usability; (b) the data transmission module that compresses and transmits the data to the cloud server taking into account the internet connection status of the watch. Due to the large volume of data produced, various lossless compression techniques were considered taking into account the limited computational and energy resources of the smartwatch; and (c) the user interface module that provides the user with basic information about the operation of the application. Analytics were also incorporated to gather critical smartwatch operating parameters such as power levels, free storage and network availability. An important aspect of the smartwatch application operation is the optimization of the computational and energy resources usage, in order to ensure adequate battery life for the project experiments. Figure 3 presents the various smartwatch application functionalities.

### 3.4. Web Portal and Data Management

Another important factor for the integrated e-Prevention platform is the web application that has been developed, allowing clinicians to access and process the patients’ personal data. Login to the dashboard is only available to the e-Prevention medical team and the involved researchers and is performed through a digitally signed authentication certificate. Each certified user has been provided with credentials granted by the e-Prevention platform. Free registration is not permitted due to security reasons since the information that is stored regarding the patients’ mental health status is sensitive and personal. Moreover, all stored information is anonymized, and the patients’ names and surnames are not registered, with the identification being performed through a unique, unchangeable identification number (ID) (thus, only the clinicians have the correspondence of patient names and IDs).

Specifically, the dashboard allows the clinicians to process the patients’ personal data, which include (see also Section 4):

(a) Demographics (e.g., age, gender, marital status, birthplace, occupation and family psychiatric history among others), recorded when a patient enters the study;
(b) Information related to the patient’s mental health diagnosis and the years experiencing it;
(c) Information regarding the patients’ medication and possible changes that occur during the course of the study;
(d) Information about various psychopathological scales, which are estimated and recorded during monthly in-person clinical assessments;
(e) Information about the relapses that each patient experienced, including their duration, severity level (low, mid or severe) and type (psychotic or not), as annotated by the clinicians.

All information described above can be updated and extracted for all or specific patients in excel and PDF file format and is handled with the help of an object-oriented relational database, which provides online backups (stored at the web portal) and ensures maximum security and data availability. The web portal is hosted on the cloud platform (Section 3.2).

**Figure 4:**
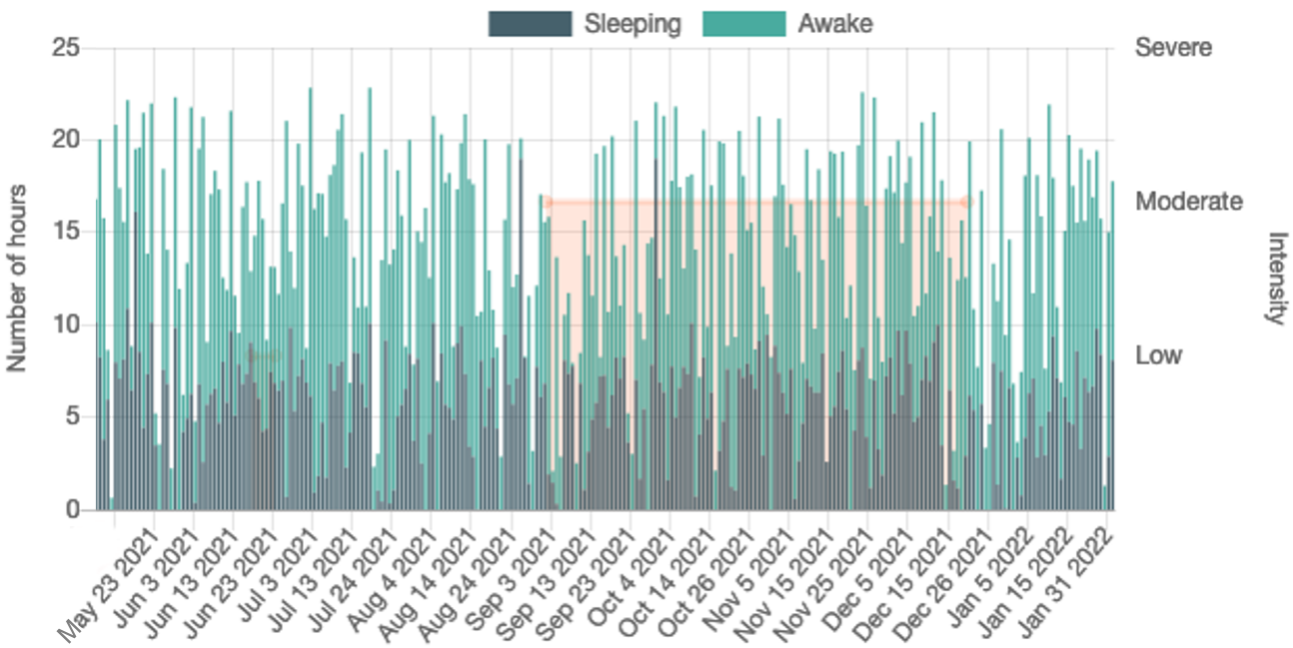
Visualization of the sleeping diary, thus the intervals during which a user was asleep or awake, along with one moderate relapse shown in the orange opaque box.

**Figure 5:**
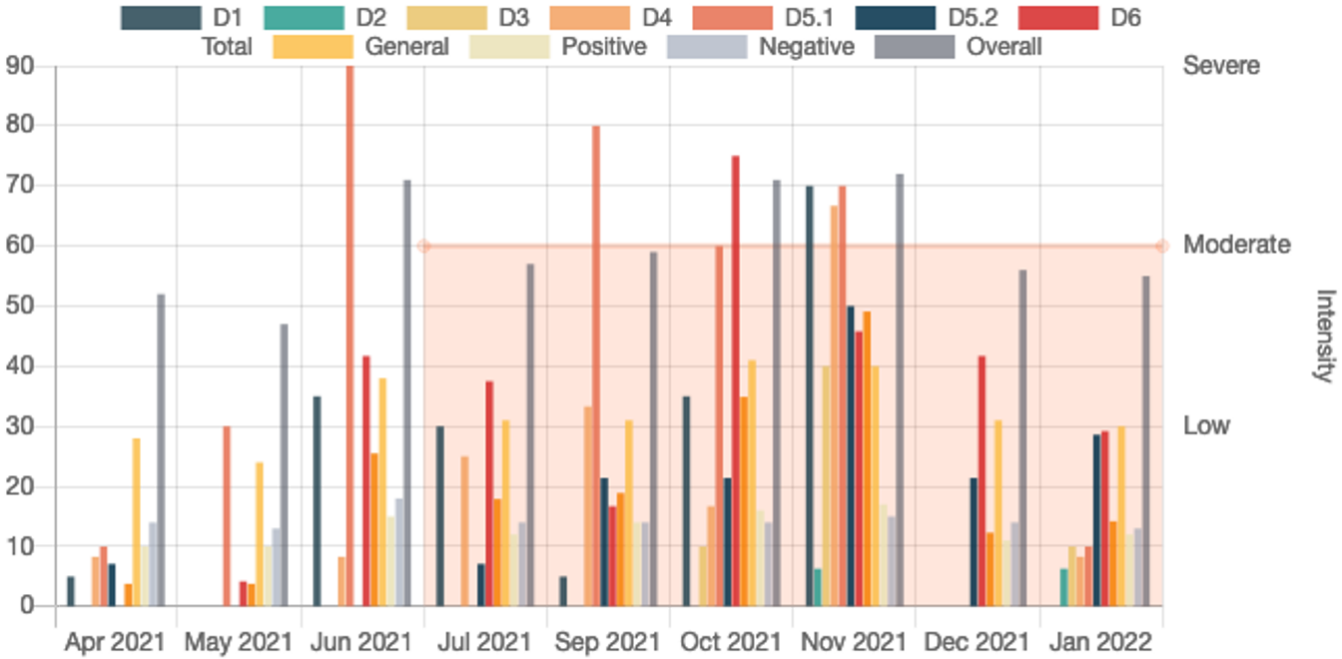
Visualization of two psychopathological scales (WHODAS II and PANSS), along with one moderate relapse shown in the orange opaque box. D1-D6 and Total refers to WHODAS II, whereas General, Positive, Negative and Overall to PANSS.

Additionally, there is a statistical data presentation functionality for each patient, including: (a) the number of hours the user was wearing the watch per day, (b) the number of hours recorded by each sensor per day, (c) a histogram of the total recorded hours per day and sensor during the user’s participation, (d) the number of hours when a user was asleep or awake per day, and (e) the number of steps in walking and running periods. The above statistics aid the clinicians to quickly access an overview of the patients’ compliance with the smartwatch usage and assist them if they do not wear it properly, e.g., wearing the watch loosely, which may lead to data loss. Figure 4 shows the visualization of the sleeping diary, while Figure 5 shows the visualization of two psychopathological scales (WHO Disability Assessment Schedule II, WHODAS II [94] and PANSS), the values of which are showing how the scales vary across each consecutive month; in both figures, a moderate relapse is displayed in the orange opaque box.

Ensuring the security and anonymity of all data is a key priority of the integrated e-Prevention system. For this purpose, specific security policies were adopted that concern the individual components but also the e-Prevention platform as a whole. International security standards for mobile applications, databases, data storage and software applications have been adopted for system security purposes.

### 3.5. Video Interview Mechanism

The e-Prevention web portal also integrates the video interview mechanism, aiding clinicians to conduct interviews with the patients from a distance, which are recorded anonymously through a dedicated tablet application. Specifically, the video interview mechanism includes the tablet application, a backend application installed on a cloud provider (Heroku), a Traversal Using Relays around NAT server and a file server. The application installed on the patient’s tablet, a Samsung Galaxy Tab A6, is used exclusively for these remote interviews, see Figure 3 for the tablet application functionalities.

The need for developing a targeted application instead of using a more commonly used calling app emerged so as to render it as easy and accessible as possible for all patients. Thus, with this application, the only thing that the patients need to do in order to start a call is to enter a specific name dictated by the clinician, which will be the same as the one that the clinician will enter through the web portal’s dashboard. The specific application was developed with Android Studio, Google’s integrated programming environment for developing applications on Android platforms, and the patients could easily download it from Google Play. A file (storage) server is used for the storage of the interview video files, which is hosted on the “∼okeanos’’ cloud server, allowing access to both clinicians and the project’s researchers to download and further process the video content.

## 4. Recruitment Protocol

Participants of the e-Prevention project, both controls and patients, were recruited at the University Mental Health, Neurosciences and Precision Medicine Research Institute “Costas Stefanis’’ (UMHRI) in Athens, Greece. Written consent and permission for use of anonymized data were given in accordance with the provisions of the General Regulation (EU) 2016/679. The protocol of the e-Prevention project was approved by the Ethics Committee of the Institution.

In the initial phase of the project, 23 participants were recruited for three months, to constitute the control group. In the second ongoing phase of the project, a total of 39 patients have been recruited up to the time of writing this paper (July 2022). Thirteen (13) patients have completed the initially agreed two-year assessment period and eleven (11) patients are still being assessed. Fifteen (15) patients have dropped out at some point during the course of their assessment, most of them for reasons unrelated to the study. Table 2 shows information about the demographics of both controls and patients up to April 2022, where we also present the amount of recorded smartwatch data for each group during wakefulness and sleep.

Before recruitment, clinicians met with the participants to conduct an assessment of symptoms and general functioning. Specifically, each volunteer underwent an initial individual assessment, lasting approximately 180 min, during which demographic data (age, sex, years of education, occupation, marital status, place of birth and residence), physical and mental health history, perinatal complications or disorders and substance abuse were examined. During the intake interview, all participants also received a neuropsychological evaluation by a trained neuropsychologist to ensure that there were not any neurological disorders. Moreover, for the control group, it was ensured that there was no history of mental disorders or substance abuse. The majority of tests administered to the patients were selected based on MATRICS (Measurement and Treatment Research to Improve Cognition in Schizophrenia) Consensus Cognitive Battery [95], which is specifically designed to assess the neurocognitive functioning of patients with schizophrenia across the following domains: speed of processing, attention/vigilance, working memory, verbal learning, visual learning, problem solving and social cognition. Verbal intelligence and verbal fluency were also assessed. Additionally, in the case of patients, a family history of mental illness was sought, along with the time (in years) from the onset of the first symptoms of mental illness, the degree of compliance with the treatment in the last 6 months and a detailed record of the medication they receive. At recruitment, the patients were in active treatment and stable. Participants were excluded if they had any of the following: (1) hearing, vision, or motor impairment; (2) a below sixth-grade reading level; or (3) inability to provide informed consent.

A number of weekly unstructured interviews of patients and controls of an average duration of 5–10 min took place through the dedicated web application developed for the e-Prevention project or through the phone in order to assess the physical activity of the participants by using the Greek version of the International Physical Activity Questionnaire—short form (IPAQ-Gr) [96]. These web interviews were recorded anonymously and stored in a secure cloud server.

Furthermore, the clinical team conducted follow-up assessments with patients once every month during which the following were assessed: psychopathology (Positive and Negative Syndrome Scale, PANSS); disability (WHO Disability Assessment Schedule 2.0, WHODAS 2.0); antipsychotic side-effects (Glasgow Antipsychotic Side-effect Scale, GASS); involuntary movement (Abnormal Involuntary Movement Scale, AIMS); extrapyramidal symptoms (Simpson-Angus Scale, SAS); and Body Mass Index (BMI) [40,94,97,98,99]. Patients also performed a computerized version of the go/no-go task that was initially administered in the neuropsychological evaluation.

In order to evaluate the emergence and severity (on a 3-point scale: low, mid, or severe) of relapses, the clinicians used the following information sources: (1) monthly assessments, which were useful for identifying the duration of the relapse, as well as its severity; (2) frequent administration of psychopathological scales; and (3) communication with the attending physician, his/her family/carer, and the hospital (in case of hospitalization). We also note whether each relapse was also classified as being a psychotic one or not. Afterwards, the clinicians inserted the information about relapses into the web portal.

## 5. Material, Methods and Research Results

In the next sections, we are going to present the most important research results that emerged during the course of the e-Prevention project. Specifically, (1) we present an extensive statistical analysis that was conducted for the identification of differences in physical activity and autonomic function patterns between controls and patients with mental disorders using both classical and novel nonlinear representations extracted from the smartwatch data (Section 5.1). (2) We continue with the implementation of autoencoder architectures for the detection and prediction of relapses using (a) physiological signals, based on the results of the previous analysis and the representations that showed statistical significance (Section 5.2); and (b) speech from interview sessions between patients and clinicians as well as a fusion between speech and physiological signals (Section 5.3). (3) Last but not least, we try to assess the patients’ symptom severity by automatically recognizing the PANSS scale, using as key indicators the patients’ facial expressions (Section 5.4). Note that due to the continuous increase in the collected data and our concurrent research, the following results are presented in different subsets of the e-Prevention collected data; thus, the reader can find the description of the used subset in each of the following subsections.

### 5.1. Statistical Analysis for the Identification of Differences in Feature Representations Extracted from Smartwatch Data

The nature of the long-term study conducted in the e-Prevention project required a different data processing approach than previous studies. For this reason, we conducted a rigorous statistical analysis in order to identify the most suitable representations in conjunction with our large dataset. Thus, inspired by traditional signal processing techniques, we extracted common and more complex features using short-time analysis and we studied them through their descriptive statistics in order to obtain a rough estimate of how they differentiate between healthy controls and patients with psychotic disorders.

The statistical analysis presented next has offered a high degree of certainty that some of both the more common and the novel nonlinear features differ extensively between the two groups, and are thus of major importance to clinical practitioners, as well as for the next steps of our experimental evaluations. Therefore, we could claim that it constitutes a vital step towards developing a method that can leverage informative and interpretable physiological and behavioral data from sensors that could act as diagnostic tools with the aim of timely prediction of relapses.

**Data Collection:** For this statistical analysis, we used data from twenty-three (23) healthy control volunteers and 24 patients with a disorder in the psychotic spectrum (12 with Schizophrenia, 8 with Bipolar Disorder I, 2 with Schizoaffective disorder, 1 with Brief Psychotic Episode, and 1 with Schizophreniform Disorder). Due to limitations on the number of available devices, each subject was recruited at a different date—controls were recruited between June 2019 and October 2019, whereas patients have been continuously recruited from November 2019 up to March 2021, when this first statistical analysis was conducted. Controls were continuously monitored for at least 90 days and then they returned the watches, whereas the monitoring of patients has been an ongoing process. In the analysis presented here, we use data up to September 2020 to ensure that the analyzed data for each group are approximately balanced. Furthermore, to mitigate the effect of the COVID-19 Pandemic quarantine in Greece (15 March 2020–10 May 2020), since only patients were monitored at the time, we exclude data collected during this period.

Table 3 contains information on the demographics of the two groups, as well as the collected data at the time of conducting the analysis. We also include the BMI and the PANSS scale rating at the time of recruitment for the two groups (note that PANSS is only applicable to patients).

**Data Preprocessing:** Short-time analysis of signals using windowing is a traditional signal processing method. In short-time analysis, we assume the process under which the data are generated to be stationary. Drawing power from these techniques, but largely increasing the time scale, we proceeded to perform “short-time’’ analysis in windows of 5 min for both movement and HRV data. Five (5) min intervals have been found to hold important information for distinguishing short-term patterns in a previous study [100].

***Preprocessing of Heart-Rate Variability:*** The heart rate variability (HRV) sequence from the 5 Hz signal was obtained by dropping identical consecutive values. We also removed RR intervals larger than 2000 ms and smaller than 300 ms, considered artifacts, and replaced possible non-detected pulses with linear interpolation. After preprocessing, we extract features from the first 4.5 min (90%) of the RR intervals sequence, dropping subsequent values so that each interval has the same length.

***Preprocessing of Accelerometer and Gyroscope Data:*** In data collected from the accelerometer and gyroscope, we first dropped all intervals with more than 50 missing values. Then, existing missing values were filled via nearest-neighbor interpolation and features were extracted from the first 5940 (99%) samples of the interval. Note also that we applied high-frequency wavelet denoising [101] in order to smooth out the intrinsic noise from the sensors. The mean and standard deviation of the number of 5-min intervals for each user is reported in Table 3.

**Figure 6:**
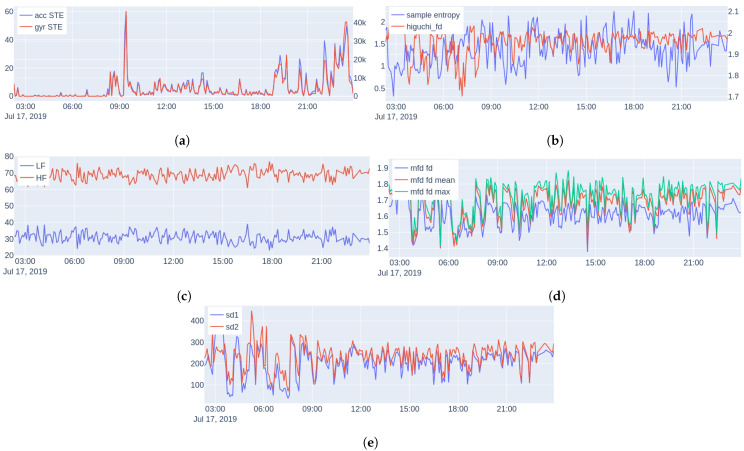
Examples of features considered in this work during one subject’s full day: (**a**) Energy (STE) of the euclidean norm of *acc* and *gyr*; (**b**) Sample entropy and Higuchi fractal dimension of HRV; (**c**) Normalized LF and HF power of HRV; (**d**) MFD fractal dimension, mean MFD and max MFD of HRV; (**e**) Poincare SD1 and SD2 of HRV.

**Feature Extraction:** We consider the following features, examples are shown in Figure 6 (during one day of monitoring a subject):

***Energy:*** The short-time energy (STE) of the euclidean norm of the accelerometer (*acc*) and gyroscope (*gyr*) signals is extracted (since they are measured triaxially). We use these features as an objective measure of physical activity and general movement behavior.

***Spectral features:*** Power Spectral Density (PSD) is a common and powerful frequency-domain method for analysis of HRV describing the relative energy of the signal’s cyclic fluctuations, managing to decompose the HRV signal to the sum of its sine and cosine components; allowing this way superimposed periodicities to be unraveled. Medical studies split the HRV spectrum into four frequency bands: ultra-low-frequency (ULF ≤0.003 Hz), very-low-frequency (VLF 0.0033–0.04 Hz), low-frequency (LF 0.04–0.15 Hz), and high-frequency (HF 0.15–0.40 Hz) [102]. Since HRV is, by definition, a non-uniformly sampled signal, we perform spectral analysis using the Lomb–Scargle (LS) periodogram [103].

The Lomb–Scargle periodogram is a method of power spectrum estimation that can be directly applied to non-uniformly sampled signals, and as a result, it is appropriate for HRV measurements. The periodogram is defined as:

(1) $$P_{LS}\left(\Omega \right)=\frac{1}{2}\left\{\frac{{\left[\sum_{n=0}^{N-1}x\left[n\right]\cos\left(\Omega \left(t_{n}-\tau \right)\right)\right]}^{2}}{\sum_{n=0}^{N-1}\cos^{2}\left(\Omega \left(t_{n}-\tau \right)\right)}\right.+\left.\frac{{\left[\sum_{n=0}^{N-1}x\left[n\right]\sin\left(\Omega \left(t_{n}-\tau \right)\right)\right]}^{2}}{\sum_{n=0}^{N-1}\sin^{2}\left(\Omega \left(t_{n}-\tau \right)\right)}\right\},$$

where τ is given by:

(2) $$\tau =\frac{1}{2\Omega }\tan^{-1}\left(\frac{\sum_{n=0}^{N-1}\sin\left(2\Omega t_{n}\right)}{\sum_{n=0}^{N-1}\cos\left(2\Omega t_{n}\right)}\right),$$

and Ω is the angular frequency (rad/s), $t_{n}$ the time (s) at which the signal was sampled, and x[n] the value of the signal at time $t_{n}$. Using the LS periodogram, we extract for each interval the normalized power in two bands: LF and HF, as well as the ratio LF-to-HF.

***Sample Entropy:*** Nonlinear methods treat the extracted time series as the output of a nonlinear system. A typical characteristic of a nonlinear system is its complexity. The first measure of complexity we consider is the sample entropy (SampEn). Sample entropy is a measure of the rate of information generated by the system and it has been considered to be an improved version of the approximate entropy [104], due to its unbiased nature.

***Higuchi Fractal Dimension:*** Multiple algorithms have been proposed for measuring the fractal dimension of time series. Here, we use the Higuchi fractal dimension [105], which has been used extensively in neurophysiology due to its simplicity and computational speed [106,107,108].

***Multiscale Fractal Dimension:*** Multiscale Fractal Dimension (MFD) is an efficient algorithm [109] that measures the short-time fractal dimension based on the Minkowski–Bouligand dimension [63]. In more detail, the algorithm measures the short-time fractal dimension using nonlinear multiscale morphological filters that can create geometrical covers around the graph of a signal, whose fractal dimension *D* can be found by:

(3) $$D=\lim_{s\to 0}\frac{\log[\mathrm{Area}\mathrm{of}\mathrm{dilated}\mathrm{graph}\mathrm{by}\mathrm{disks}\mathrm{of}\mathrm{radius}s/s^{2}]}{\log(1/s)}.$$

As is known, *D* is between 1 and 2 for one-dimensional signals, and the larger the *D* is the larger the degree of geometrical fragmentation of the signal. In practice, real-world signals do not have the same structure over different scales; hence, *D* is computed by fitting a line to the log–log data of Equation (3) over a small scale window that can move along the *s* axis creating a profile of local multiscale fractal dimensions (MFDs) D(s, t) at each time location *t* of the signal frame; thus, we are able to examine the complexity and fragmentation of the signals at multiple scales. In general, the short-time fractal dimension at the smallest discrete scale (s=1) has been found to provide some discrimination among various events. At higher scales, the MFD profile can also offer additional information that could help further the discrimination; more details about the algorithm can be found in [109]. For this reason, we summarized the short-time measured MFD profiles by taking the following statistics: fd[1] (the fractal dimension), min, max, mean, and std for each 5-min HRV interval.

***Poincare Plot Measures:*** The Poincare plot [61] is a recurrence plot, where each sample of a time series is plotted against the previous, and then an ellipse is fitted on the scatter plot. The width of the ellipse (SD1) is a measure of short-term HRV, whereas the length (SD2) is a measure of long-term HRV.

***Feature Aggregation:*** Using the information from the sleep schedule of each subject, we split the intervals into two groups; one corresponding to intervals during sleep and one during wakefulness. We then calculated the mean and standard deviation (std) over all individual intervals, resulting in two values for each subject and feature type and a total of 28 features. Significance tests using the Student’s *t*-test showed no significant differences between the recorded movement and HRV intervals for each group and state (i.e., sleeping and awake). Normality was tested with the Shapiro–Wilk test [110].

**Figure 7:**
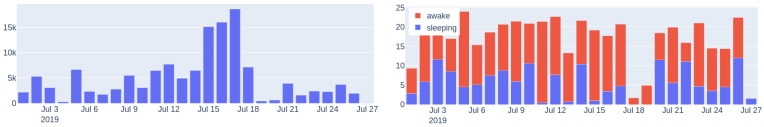
Steps per day (**left**) and hours spent sleeping and awake (**right**) during one month of a subject’s recordings.

***Sleep/Wake Ratio and Steps:*** In addition to the above features, we also extracted for each subject the mean and standard deviation of his/her sleep/wake ratio and the mean number of steps per day. Since the number of recorded hours each day fluctuates, for these features, we use only days with at least 20 recorded hours (no significant difference found between the number of days for controls and patients using Mann–Whitney U testing [111], since the normality assumption was violated). Figure 7 shows the steps per day and sleep/wake cycle during one month of monitoring.

**Figure 8:**
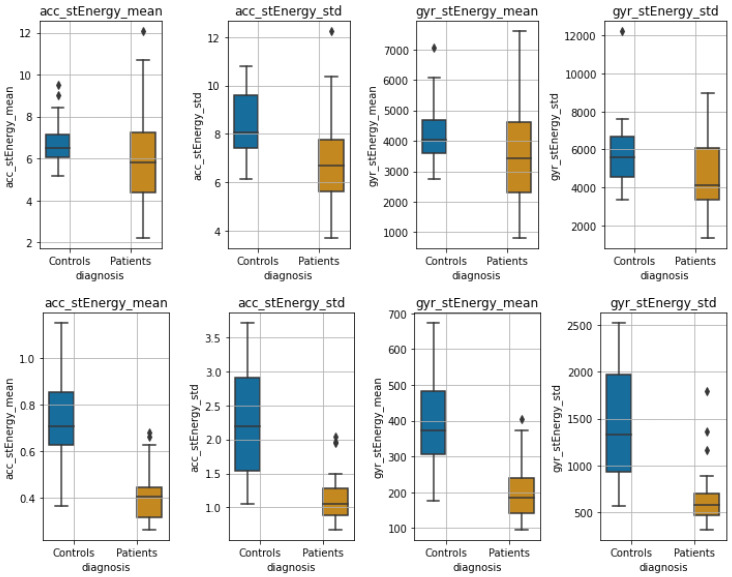
Boxplots for accelerometer and gyroscope features of controls (in blue) and patients (in light brown) while (**top**) awake and (**bottom**) asleep. The bold line represents the median, the boxes extend between the 1st and 3rd quartile, whiskers extend to the lowest and highest datum within 1.5 times the inter-quantile range (IQR) of the 1st and 3rd quartile, respectively, and outliers are shown as diamonds.

**Figure 9:**
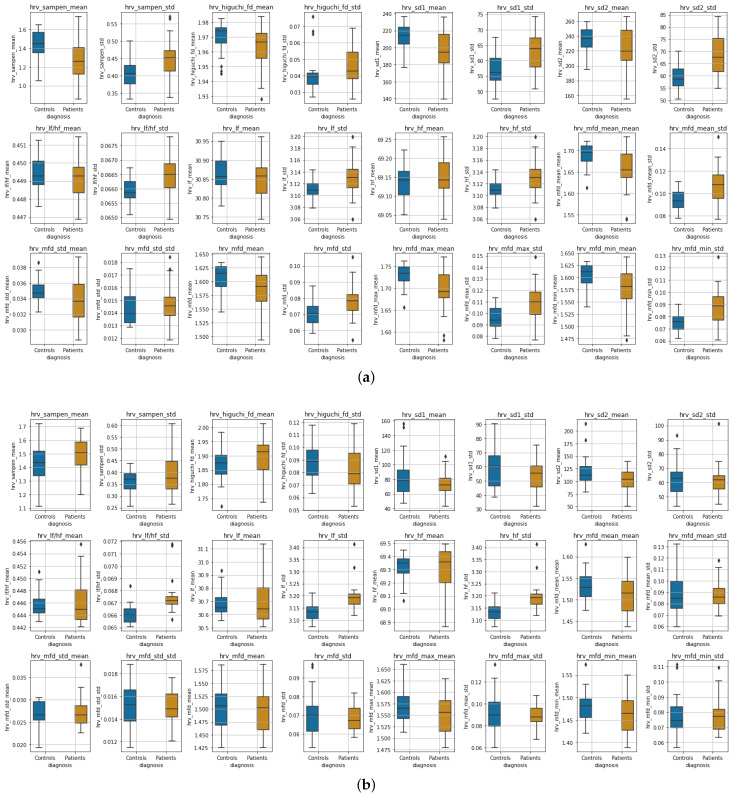
Boxplots for heart rate variability features of controls and patients while awake (top rows) and asleep (bottom rows). The bold line represents the median, the boxes extend between the 1st and 3rd quartile, whiskers extend to the lowest and highest datum within 1.5 times the inter-quantile range (IQR) of the 1st and 3rd quartile, respectively, and outliers are shown as diamonds. (**a**) Awake; (**b**) Sleeping.

**Experimental Results:**Figure 8 shows boxplots of the features extracted from the accelerometer and gyroscope data during wakefulness and sleeping, whereas in Figure 9, boxplots of the HRV features are presented for the two states, respectively. Due to the differences observed perceptually between the distributions in most features, we tested for significant differences between distributions (the null hypothesis being that the two distributions are the same) using two-tailed non-parametric Mann–Whitney U tests [111]. We adjusted for *p*-values using the Benjamini–Hochberg (BH) procedure [112]. Due to the nature of our study, BH was preferred over the more strict Family-Wise Error Rate methods [113]. Table 4 shows the results of Mann–Whitney U tests for all features.

***Wakefulness Comparison:*** During wakefulness, the features that pertain to movements appear to present more variability in the patient group compared to the controls, as shown in Figure 8 (top row). The same appears to also be valid for some nonlinear HRV features, for instance, SampEn mean, Higuchi mean and std, SD1 and SD2, various statistics extracted from the MFD profile, and some of the frequency domain features, as can be seen in Figure 9 (three top rows). Additionally, the significance testing presented in Table 4 showed significant distribution differences in the *std* of *acc* and *gyr* short-time energy, the *mean* and *std* of SampEn, the *std* of SD1 and SD2, the *std* of LF, HF and LF-to-HF ratio as well as the MFD statistics related to *std* as well as to *mean*, *max* and *min std*. The other features failed to reject the null hypothesis.

***Sleep Comparison:*** Similarly, Figure 8 (bottom row) presents the accelerometer and gyroscope feature distributions for each group during sleeping, whereas Figure 9 (three top rows) shows the distributions of HRV features. It is evident that especially the movement-related features present a significant difference, which is also verified in the Mann–Whitney U test results shown in Table 4. The mean of the sample entropy among others also appears to be different (large variations); however, the null hypothesis could not be rejected, possibly due to *p*-value adjustments for multiple testing. From the rest of the features, the *std* of LF, HF, and their ratio were found to differ significantly.

**Figure 10:**
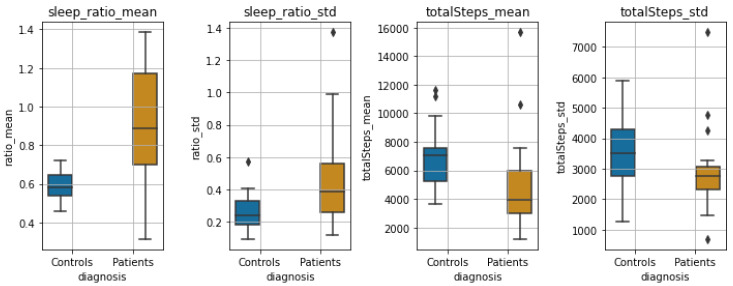
Boxplots of sleep/wake ratio and steps per day (mean-std).

***Sleep-wake Ratio and Total Steps:*** Finally, Figure 10 shows the boxplots of the statistics of steps per day and sleep/wake ratio for the two groups. We observe a large significant difference between both the distributions of the *mean* and *std* of the sleep/wake ratio (p<0.001 and p=0.01, respectively), as well as the mean and std of total steps per day (p=0.01 and p=0.05, respectively).

**Discussion:** Our goal with the statistical analysis is to exploit various traditional but also less-known and, at the same time, more novel signal processing techniques to identify common markers/features that differ drastically when a person has a psychotic disorder. These markers could prove useful in predicting potential relapses in these patients.

Our findings have shown that patients tend to behave with greater variability and present large outliers—some behave close to controls, whereas others might show extreme values. During wakefulness, even though the mean energy did not differ when compared to controls, the standard deviation showed a significant difference, indicating that patients tend to depict large variations in their movement behavior. On the contrary, during sleeping, the patients presented a small mean and standard deviation of the energy in each of their sleeping intervals compared to the controls. We should note, however, that the observed differences in sleep between the two groups could be attributed to medication administered to patients, which possibly causes variability in sleep duration.

Some of the nonlinear features that were measured for the HRV data showed significant differences in the distributions between controls and patients, i.e., during wakefulness; as seen in Table 4, such features are the mean and standard deviation of the sample entropy, as well as various statistics derived from the MFD analysis. Furthermore, the standard deviation of the normalized low- and high-frequency bands of the HRV, as well as their ratio, were found to differ significantly both during wakefulness and sleeping. During sleeping, we did not find any other measurements of HRV to differ significantly. Finally, the sleep ratio of the two groups, as well as the mean and std of the number of steps per day, presented significant variation between the two groups.

The main merits of this statistical analysis are two-fold: First, compared to previous similar studies, which have mostly lasted for a few weeks, our study, at the point that was conducted, had already been going on for more than a year. To do this, we employed a commercial off-the-shelf smartwatch that had been acknowledged by our volunteers to be comfortable, and patients are willing to insert it into their daily lives routine. Second, we show how traditional short-time analysis combined with common but also more complex and novel features, such as the MFD features that depicted significant differences in wakefulness data, can be employed to identify biomarkers and present large inter-group variabilities between healthy controls and patients, paving the way towards both acquiring clinical insights on psychotic disorders, but also exploring the capabilities of these markers to predict relapses. In the next Section 5.2, we present our work on relapse detection, using some of the features found statistically significant in this analysis.

### 5.2. Relapse Detection Using Smartwatch Data and Autoencoder Architectures

In order to detect relapses in patients with psychotic disorders using the physiological signals collected by the smartwatch, we followed an anomaly detection approach [89,114]. In particular, we examined four different autoencoder architectures based on Transformers, Fully connected Neural Networks (FNN), Convolution Neural Networks (CNN) and Gated Recurrent Units (GRU) [115,116], with our models implemented using both a personalized and a global scheme. For this task, we used time-scaled data of 1569 days, segmented into five-minute intervals, from ten patients, obtaining encouraging results. We also conducted an analysis using the best-performing models to examine the ability to estimate the severity level of a relapse among patients who relapsed multiple times with different severity levels, providing important evidence as well [91].

**Data Collection:** At the time that this study was performed 24 patients with a disorder in the psychotic spectrum had already been recruited; however, after the preprocessing of the raw data (and taking under consideration missing data that could not be recovered), we were able to process only data from 10 patients, of which 2 with Schizoaffective Disorder, 4 with Bipolar I Disorder, 1 with Brief Psychotic Episode, 1 with Schizophreniform Disorder and 2 with Schizophrenia; see Table 5 for demographics. Specifically, the data were collected from November 2019 to September 2021, with the exact period varying for each patient due to the differences in time of recruitment; after preprocessing, the data amounted to a total of 1569 days. Depending on the clinician annotations, as described in Section 4, we split the data into three categories: normal data, where the patient was stable; relapse data corresponding to time periods when a relapse had occurred; and near-relapse data, thus data recorded up to 21 days prior to the appearance of each relapse. For this study, we discard near-relapse data and keep data corresponding to stable and relapsing periods.

**Feature Extraction and Data Preprocessing:** As the first step in our analysis, and based on the statistically significant representations as described in Section 5.1, we performed feature extraction, including in our experiments the following features: the mean energy of the accelerometer and gyroscope norm, the mean heart rate and R-R interval, the normalized energy in the LF and HF bands of the heart rate (0.04–0.15 Hz and 0.15–0.40 Hz, respectively), and the value of the width of the ellipse in the Poincare recurrence plot. Moreover, three additional features were included to model the chronological order of the time-series and how well the patient was wearing the watch, specifically, the sine and cosine representations of the corresponding seconds (over a daily period) and the percentage of correctly identified pulses in the given interval.

Afterwards, the features were aggregated into a dense representation of 5-min intervals, since as shown in [100] such intervals are able to capture micro-scale patterns, something that also allowed us to have an adequate amount of data for the deep learning architectures that we implemented. These intervals were then stacked temporally into tensors, each covering 24 h of physiological activity. In cases of missing data up to 10 consecutive hours (e.g., when the patient was charging or did not wear the watch), we filled the data with the median values of the missing feature over a temporal window; when more than 10 consecutive hours of data were missing, we completely disregarded the specific interval.

**Methodology:** We implemented four different architectures based on autoencoders that learn to reconstruct an input time series; specifically, Transformers, Fully connected Neural Networks (FNN), Convolutional Neural Networks (CNN) and Gated Recurrent Unit (GRU), see also [91].

In the Transformer model, the input sequence is first imported into a positional encoding layer followed by four stacked transformer encoder layers, which are made up of two sub-layers. Both sub-layers are followed by a normalization layer. The input sequence after the encoding is reversed and piped into the decoder, which consists of four decoder layers, with similar architecture to the encoder. At the end, a linear layer is applied.

For the Gated Recurrent Unit (GRU) sequence-to-sequence model with attention, a subsequence of data is fed into the encoding layer of the GRU with a specified hidden unit of size 100. Afterwards, we pass the weighted average of all encoded outputs (attention vectors) from all time-steps as inputs into the GRU decoder layer that reconstructs the subsequence.

The Fully connected Neural Network autoencoder (FNN) encompasses 2 fully connected encoder and decoder layers that compress an input subsequence to a lower dimension in order to reconstruct the initial subsequence. A ReLU nonlinearity follows after each fully connected layer and the last layer also contains a dropout layer in order to avoid over-fitting.

The CNN-based autoencoder follows an encoder-decoder scheme, with the encoder mapping the input to a low-dimensional latent representation, and then the decoder trying to reconstruct the original input. We build the decoder using 4 downsampling blocks which consist of an 1D Convolutional layer, batch normalization, and a LeakyReLU activation. In a similar manner, we build the decoder using 4 successive upsampling convolutional blocks, mirroring the blocks of the encoder. Finally, we apply a linear layer at the top.

**Training and Evaluation of Anomalies:** Models were trained using both a personalized and a global scheme. In the **personalized scheme** the evaluation is performed for each patient separately, which is a common and proposed procedure for such tasks [46]. However, we wanted to explore the generalization capabilities of our methods, so we evaluated them using a **global scheme**, as well; thus, we train our models on data corresponding to all patients. For each case, we separated the respective normal data into three sets, i.e., the train (60%), validation (20%) and test (20%) set, following a 5-fold cross-validation scheme; the validation set was internally split into two equal subsets. All data were normalized in the [0,1] range, apart from the sine and cosine time representations, which were already in [−1,1]. The train and validation sets, as usually performed in such anomaly detection tasks, contained only data with no anomalies (i.e., “normal” data), whereas the test set contained data both without and with anomalies (thus, relapses). Consequently, the performance of all models was evaluated to “unseen” normal and relapse data.

For the architecture’s implementation, we used Pytorch and the Mean Square Error (MSE) between the reconstructed and input time-series as a loss function for training (batch size of 64). For the Transformer and the FNN AE, the Adam optimizer [117] was used, whereas the RMSprop optimizer was used for CNN AE and GRU AE. All models had a learning rate equal to 0.0001, with the exception of the Transformer having 0.001. The training took place for 50 epochs, and early stopping was applied to monitor the model’s performance, using the validation loss of the first validation subset.

The mean absolute error (MAE) between the predictions ${\hat{x}}^{\left(i\right)}$ and the given data $x^{\left(i\right)}$ was calculated in order to obtain the reconstruction error vector with size l×d of each point *i*. The error vectors $e^{\left(i\right)}$ in the second validation subset are used to compute the mean (μ) and covariance (Σ) of a multivariate normal distribution that is the expected error distribution. Afterwards, the “anomaly score” was computed as the Mahalanobis distance between the predicted points in the test set and the Gaussian distribution that was calculated in the respective validation subset as in [118]:

(4) $$a^{\left(i\right)}=\sqrt{{\left(e^{\left(i\right)}-\mu \right)}^{T}\Sigma^{-1}\left(e^{\left(i\right)}-\mu \right)}.$$

The per-point anomaly scores are day-averaged similarly to [48]. The performance of each architecture was evaluated by the Receiver Operating Characteristic Area Under Curve (ROC AUC) and Precision-Recall Area Under Curve (PR AUC) metrics. Since we utilize 5-fold cross-validation, the median metric over all folds is reported as the final score. Concerning the evaluation of global models, we evaluated them either *globally* (global scheme, tested to all patients) or *individually* (global scheme evaluated individually, thus per patient).

**Experimental Results and Discussion:** In order to obtain baseline results, we implemented a random classifier (referred to as Random in Table 6, Table 7 and Table 8), where we classified the data without training the models. Specifically, we directly calculated the mean and the covariance of the features in the validation set and then the anomaly scores in the test set. Table 6 and Table 7 show the results of our experiments for the personalized scheme for all patients and models. The best results for each patient are shown in bold. We observe that the best overall performance is obtained by the CNN AE model, with a PR-AUC equal to 0.76, whereas all personalized models’ results surpass the baseline.

**Figure 11:**
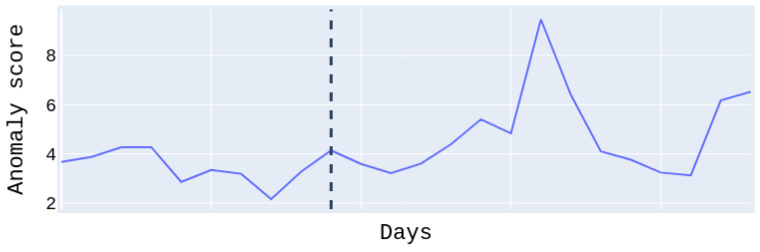
Anomaly score of Patient #1. Note that the Days on the x-axis are not continuous.

Figure 11, shows the anomaly score of the test set for patient #1, who suffered a moderate relapse of about 11 days. The anomaly score to the right of the dividing line regards the relapse days and to the left the normal days. We easily notice that the anomaly score during relapse days is higher than on normal days. Note that the days on the *x*-axis are not continuous. For this patient, we obtained the best PR and ROC AUC scores at 0.97 with the Transformer model. We have to emphasize at this point, that for some patients the performance is low. This is due to the fact that there was only a low amount of available relapse data (in some cases only a few days or hours after preprocessing); nonetheless, they were not excluded (as possible outliers), in order to maintain an adequate amount of data for the experiments. However, the conducted *t*-tests showed statistically significant improvement for six out of ten patients over the random baseline, with a *p*-value lower than 0.05, whereas patients with more relapse days and data, i.e., patients #6 and #7 had better results related to the tests.

A number of ablations were performed on the best-performing architecture, namely the CNN AE, concerning the type of features utilized as well as the temporal dimension of the input tensors. Particularly, in Table 9, we present the median per-patient ROC-AUC and PR-AUC scores obtained by discarding either the features from the accelerometer and the gyroscope or those related to the heart rate. We observe that both types of features are necessary to achieve a good performance; using only the heart-rate-based features, we obtain a similar ROC-AUC score but a lower PR-AUC, whereas the drop in the PR-AUC is more noticeable when using only features from the accelerometer and the gyroscope.

**Figure 12:**
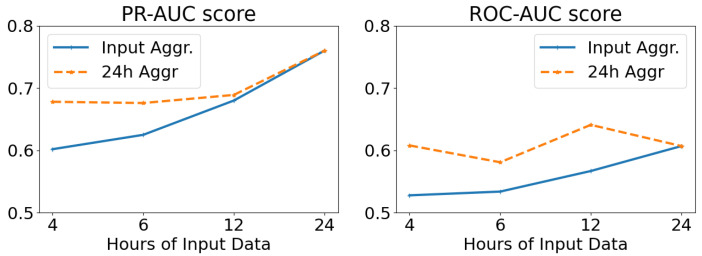
Ablation study on the effect of the length (duration covered in hours) of the input tensors and the aggregation period, for the CNN AE architecture.

The results of altering the length (in hours) of the network input, while keeping the temporal resolution stable (and equal to 5-min intervals), are displayed in Figure 12. We examine averaging the results at the temporal window of each input tensor (Input Aggr., ranging from 4 to 24 h), as well as on a daily basis irrespective of the input length (24 h Aggr.). From these results, we deduce that the larger the length of the network input, and thus the temporal window of analysis, the better the overall predicting ability of our model. We also note that per-day aggregation of the anomaly scores yields better results than using the temporal dimension of the input as a scale.

Table 8 presents the results for the global scheme and the global scheme evaluated individually. In this case, the FNN AE model, which was evaluated individually (denoted as Median), had the best performance with PR and ROC AUC of 0.77 and 0.62, respectively, whereas the global scheme (denoted as Global) in general presents lower performance than the models that were evaluated individually, possibly due to the fact that several of the relapses had “low” severity, thus making their detection harder. Another important observation that we draw is that patients with moderate and severe relapses yielded better PR and ROC AUC results than the others.

Finally, using the best-performing models we experimentally evaluated the importance of relapse severity, using data from three patients that relapsed multiple times with different severity levels (Low and Moderate), while also examining the differences presented between low, moderate and severe relapses across all patients and severity level. For the former, we observed that for patients with adequate data the reconstruction error for moderate relapses was higher than the one recorded for the low-severity relapses, whereas for the latter, as intuitively expected, we noted that there was a gradual increase in the reconstruction error in relation to the severity of the relapse.

### 5.3. Relapse Detection and Prediction from Spontaneous Speech

Apart from physiological data, we investigated the extent excerpts from the spontaneous speech of the patients can be used to either detect relapses or predict their appearance. We opted for an unsupervised learning approach since it provides the advantage that models can be trained without necessarily having access to data from relapsing periods. Experiments conducted in a database with a total of 16 patients, containing over 38,000 s of total speech, yielded encouraging results for both classical Convolutional Autoencoders (CAEs) and Convolutional Variational Autoencoders (CVAEs) in a personalized setting, in agreement to our previous results derived from smaller subsets of this database [86,119]. Moreover, CVAEs can reach the performance of personalized models in a global (patient-independent) setup, especially when per-person normalization is applied to the input features [119]. Finally, we experimented with a decision-level fusion between audio and physiological data, with the results indicating that physiological signals can act as a complementary modality to audio.

**Data Collection and Preprocessing:** For the purposes of this set of experiments, we utilized the short interviews between the patients and the clinicians, recorded through the e-Prevention app. Since not all patients recorded interviews, for the subsequent experiments we used interview data from 16 patients (1 with Schizoaffective disorder, 1 with Schizophreniform disorder, 1 with Bipolar II disorder, 8 with Schizophrenia and 5 with Bipolar I disorder), collected between May 2020 and December 2021. Patient demographics used in this work are presented in Table 10. Eight (8) out of the sixteen patients had experienced a relapse during the duration of this study, whereas the rest were selected according to the total duration of their interviews. Each interview was annotated, according to the condition of the patient at the time it was conducted. In particular, interviews were split into clean data (C), where no relapse had been detected by the clinicians, relapse data (R), including interviews where the patient’s condition was denoted as relapsing, and pre-relapse data (P), which correspond to time periods up to 30 days prior to the appearance of a relapse. For the purposes of this work, both relapsing and pre-relapsing data were considered anomalous. Note that in contrary to the work described in Section 5.2, using only smartwatch data, the period for pre-relapse data is different by 9 days; however, both duration of pre-relapse days that were selected are valid and were dictated by the clinicians of the e-Prevention project.

These interview videos were then preprocessed, in order to facilitate the feature extraction. In particular, the audio excerpts were extracted from the interviews and downsampled to 16 kHz. In order to isolate the utterances corresponding to patients from the complete audio excerpts, the x-vector [120] diarization recipe from kaldi [121] was used. This process resulted in a total of 14,562 utterances (38,066 s), about which we present detailed statistics in Table 10. For each utterance, the log mel-spectrogram was computed, using a frame length of 512 samples (approx. 30 ms), an overlap of 256 samples (approx. 15 ms), and 128 mel bands. Finally, these spectrograms were cut off along the time axis in slices of 64 frames (approx. 1 s), thus yielding a 128×64 representation for each second of speech.

For the experiments examining the effect of fusion between audio and physiological information in detecting and predicting relapses, it was necessary to produce day-aligned pairs of interviews and physiological data. To ensure the availability of an adequate amount of paired data for each patient, these experiments were conducted with a reduced set of 12 patients (1 with Schizoaffective disorder, 1 with Schizophreniform disorder, 7 with Schizophrenia, 2 with Bipolar I disorder and 1 with Bipolar II disorder), out of whom 6 had experienced a relapse during the course of this study. Thus, taking into account the coupling of modalities, 164 interview sessions, containing 5623 utterances with an overall duration of 14,534 s, and 2233 h of physiological data were used. In order to deal with the reduced amount of physiological data, compared to Section 5.2, the respective features (in this case, the mean heart rate and R-R interval, the peaks of the high frequency (HF, 0.15–0.4 Hz) and low frequency (LF, 0.04–0.15 Hz) bands of the Welch periodogram, and the ellipse width of the Poincare recurrence plot) were averaged over 2-min intervals, and then stacked temporally into a 5×30 representation, covering one hour of physiological activity.

**Architectural Details:** In the case of the CAE model, the encoder contains 4 convolutional blocks, which progressively reduce the dimensionality of the input mel-spectrograms to produce a low-dimensional embedding. Each block consists of a 2D-Convolution layer with an increasing number of filters at each block, a ReLU activation function, and a 2D Max Pooling layer. In turn, the decoder restores the latent embedding into its original dimensionality by applying a series of 4 upsampling convolutional blocks upon it. These blocks alternate upsampling layers with 2D-Convolution layers, followed by ReLU activations with the exception of the last layer. The reconstruction objective was enforced through an MSE loss ($L_{MSE}$) between the true and estimated spectrograms.

The CVAE is built upon the CAE architecture presented above, but instead of compressing its input into an intermediate latent representation, the encoder of the CVAE learns a $\left(\mu ,\sigma^{2}\right)$ Gaussian distribution, from which embeddings are sampled and decoded through the decoder. To this end, the last convolutional block of the encoder is replaced by a pair of parallel convolutional blocks, which estimate the parameters of the latent Gaussian distribution. This distribution is encouraged to align with the spherical isotropic Gaussian, N∼(0,I), through the imposition of a Kullback–Liebler divergence loss ($L_{KL}$) in the bottleneck of the network. For more details about the developed architectures, we refer the reader to [86,119].

**Experimental Protocol:** Concerning relapse detection and prediction from audio data, we trained models for both the personalized and the global case. In the personalized case, a separate model is trained for each patient who had experienced a relapse during the course of the study, using their respective speech segments. On the other hand, in the global case, a single model is trained for all patients, regardless of whether they had undergone any relapses, using the whole set of interview data. In both cases, we followed a 5-fold cross-validation training protocol, where the networks were trained only using clean data, and evaluated on a mixture of clean and anomalous data. In particular, for each fold, the clean data were split into training (60%), validation (20%) and testing (20%) data. The clean testing data were then merged with the anomalous (corresponding to pre-relapsing or relapsing states) data, in order to form the evaluation set. We further note that to avoid session-wise overfitting, data were split so that spectrograms corresponding to the same interview belong in the same fold. All networks were trained for a maximum of 200 epochs, with early stopping being applied with a patience of 10 epochs. The networks were optimized using Adam [117] with a learning rate equal to 3e−4 and a batch size equal to 8, and the loss weights for the case of the CVAE were set as $W_{MSE}=1$ and $W_{KL}=0.01$, respectively. We also note that to ensure the robustness of the results, each experiment was repeated five times, and we report on the average of the results over all repetitions.

The performance of both architectures is evaluated on a per-session basis. Each spectrogram provides an anomaly score; in the case of the CAE, it is derived by the reconstruction error, whereas in the case of the CVAE, we investigate the suitability of both the reconstruction error of the spectrogram and the KL divergence of its projected representation. A single value of the anomaly score for each session is then computed by temporal aggregation of the per-spectrogram scores. We utilize as our evaluation metrics the median anomaly score over all sessions, according to the state of the patient and the mean ROC-AUC score over all folds, applied to the per-session anomaly scores. We also perform an ablation study on the temporal pooling functions used to aggregate the per-spectrogram anomaly scores into a single anomaly score for each session. Finally, we investigate the effect of per-patient normalization of the features in the global case, as opposed to global feature normalization.

Experiments concerning the fusion of audio and physiological signals were only conducted for the global case. In this case, a pair of unimodal neural networks were trained independently and then evaluated individually in coupled speech from interview sessions and physiological signals from the day the interview was conducted. The unimodal anomaly scores were temporally aggregated, similar to above, on a per-session basis and a daily basis, respectively, and then combined using decision-level fusion. To this end, we experimented with two fusion mechanisms, namely additive (Add.) and multiplicative (Mult.) fusion.

**Personalized Experiments:** In Table 11, we present the medians of the per-session anomaly scores for each patient, depending on the state of the patient during the interview session (clean, pre-relapsing or relapsing) and the network configuration used for deriving the anomaly score. We observe that for the majority of patients, interviews corresponding to either pre-relapsing or relapsing states record higher anomaly scores compared to those conducted when the patient’s condition was annotated as stable. Interestingly, this trend appears to be more profound for interviews conducted in pre-relapse periods, than during relapses. We finally note that when the KL divergence was used as an anomaly measure, the anomaly scores exhibit lower variability among different patients compared to the reconstruction error, implying its potential scalability to a subject-independent setting.

In Table 12, we report on the macro-average of the ROC-AUC score for each network configuration, according to the pooling function used to aggregate the per-spectrogram anomaly scores into a single measure, and considering both pre-relapsing and relapsing states as anomalous. With regard to the temporal pooling function, we compare average pooling (AP), max pooling (MP) and norm pooling (NP) [122] using the fixed value p=10. No statistically significant deviations between the evaluated models were found at the p=0.05 level, after using the Bonferroni-corrected Mann–Whitney U-Test. We observe that when using the reconstruction error as the anomaly score, average pooling performs best, whereas comparatively better results are acquired when using the norm pooling for temporal aggregation of the KL divergence scores of the CVAE. Finally, the per-patient ROC-AUC scores when using the best performing pooling function for each model are presented in Table 13, where we observe that for five out of the eight patients, a ROC-AUC score higher than 0.7 is achieved.

**Global Experiments:** In Table 14, we present the medians of the per-session anomaly scores for all patients, depending on their state during the interview and the network configuration used, as well as the application of global or per-patient normalization. We observe that when using global feature normalization, pre-relapsing sessions record only slightly higher anomaly scores than sessions conducted under stable patient conditions, whereas lower scores are acquired from sessions corresponding to relapsing states. On the other hand, per-patient normalization leads to better discriminability between clean sessions and sessions corresponding to both anomalous states in the case of the CVAE. The trend observed in the personalized case, regarding the higher anomaly scores yielded by pre-relapsing sessions compared to ones recorded during relapsing periods, is noticeable in this case as well.

The ROC-AUC scores depending on (i) whether features were normalized globally or per-patient, and (ii) the temporal pooling function used to aggregate the per-spectrogram anomaly scores are presented in Table 15. These results support the qualitative claims deduced from the median anomaly scores presented above. Namely, when the input spectrograms are normalized per patient, and the KL divergence is used as the anomaly score, the global CVAE-based model reaches comparable performance to the personalized models, outperforming the original CAE. On the other hand, the application of global normalization to the spectrograms does not lead to performance improvements over random chance, irrespective of the model used. The necessity of per-patient normalization as a means to reach the performance of a personalized model is also consistent with [72].

Concerning the suitability of the temporal aggregators evaluated, in contrast to the personalized case, average pooling yields the worst performance, whereas a global ROC-AUC score of approximately 0.7 is reached when using norm pooling as the aggregator, in conjunction with the KL divergence of the CVAE as a scoring function. Indeed, application of the Mann–Whitney U-Test to the ROC-AUC scores (over all folds and experiment repetitions, N=25) indicates the superior performance of the KL-CVAE over the other two configurations at a p=0.05 statistical significance level post-Bonferroni correction, irrespective of the temporal pooling function and the normalization protocol used. The superior performance shown by the CVAE, compared to the CAE, in the global setup is in agreement with [123], where VAE-based models were used to successfully extract speaker-invariant features from speech signals, indicating decreased dependency on person-specific speech properties.

**Figure 13:**
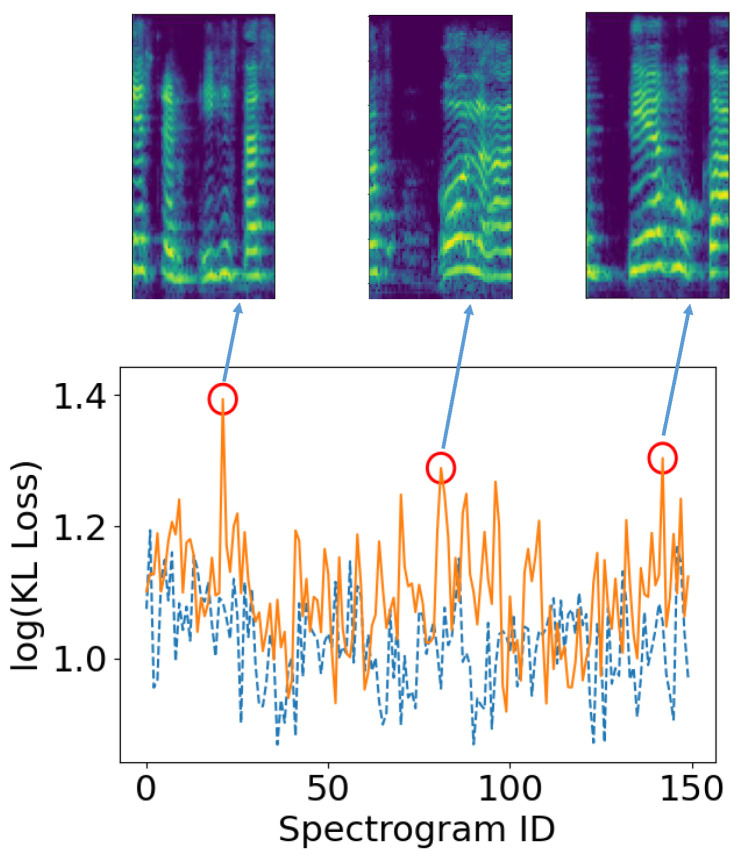
Per-spectrogram visualization of the KL divergence scores for two interview sessions of the same patient, one corresponding to stable (dashed blue) and one to relapsing (orange) condition.

In Figure 13, we present the per-spectrogram KL loss for two interview sessions corresponding to the same patient. We observe that the spectrograms that correspond to stable (dashed blue) patient conditions do not exhibit consistently higher anomaly scores compared to those comprising the interview recorded during a relapse (orange). However, a number of peaks appear in the anomaly scores of the relapsing session, denoted with red circles. Upon examination of the speech excerpts, whose spectrograms are also displayed in Figure 13, we observe that they correspond to segments with abrupt pauses in the patient’s flow of speech.

**Multimodal Fusion:** Before presenting the results on the fusion between audio and physiological data, we evaluate a number of modifications to the CNN-AE architecture presented in Section 5.2 operating on the physiological data to make it suitable for a global (patient-independent) setup. Since the results presented above indicate that, in conjunction with a per-patient feature normalization scheme, CVAEs can scale in a global relapse detection and prediction setting from audio better than CAEs, we examine the effect of those adjustments to the CNN-AE using physiological signals. In particular, we adapt the CNN-AE architecture into a 1D-CVAE following a similar procedure to the one used for the speech-based architecture, while also examining the effect of per-patient normalization on the input physiological tensors. Concerning the per-instance anomaly score, we investigate as potential probe points either the Mahanalobis distance between the predicted error distribution and the error distribution of the validation set [118], denoted as EMD, or the KL-divergence of the input embeddings.

The results are provided in Table 16, leading to similar conclusions to those acquired from the speech segments. Namely, the per-patient normalization appears to positively affect the performance of the network, irrespective of the architecture used, whereas using the 1D-CVAE with the KL divergence as an anomaly score of the HRV tensors leads to improved performance compared to the original CNN-AE. Thus, based on these results in conjunction with those of the previous sections, networks for both modalities are realized as appropriately designed CVAEs, with the input features of the respective modality being normalized per subject and the anomaly scores, for both modalities, being derived from the KL divergence of the respective embeddings.

The results acquired from combining audio and physiological data using this configuration are presented in Table 17; apart from the two fusion mechanisms we examine, we present the results acquired by only using a single modality. We observe that using both modalities with either fusion mechanism yields higher results than using only a single modality, indicating that physiological signals can be utilized as auxiliary information in speech-based relapse detection and prediction. Concerning the fusion mechanisms, additive (Add.) fusion appears to provide slightly improved results compared to multiplicative (Mult.) fusion.

We also examine the effect of the temporal pooling mechanism used to aggregate the anomaly scores for each modality; we report on the results in Table 18. Similar to the unimodal audio dataset, norm pooling provides the best results concerning the per-session aggregation of the anomaly scores for each spectrogram. However, regarding the physiological data, we found that daily averaging of the per-hour scores resulted in the best performance. In fact, usage of the other two potential temporal aggregation functions negated the benefits of the multimodal relapse detection scheme, resulting in performance lower than the one of the unimodal audio CVAE.

### 5.4. Evaluating Mental Conditions Utilizing Facial Cues from Videos

Finally, in this section, we describe our work in trying to predict PANSS indicators related to facial cues, using both handcrafted and learned features, extracted from the videos of the unstructured interviews conducted between patients and clinicians. Indeed, facial expressions of patients could be a key indicator towards the quantification of cognitive impairments [124], and recent advancements in computer vision, machine and deep learning allow the evaluation and recognition of such temporal emotional status, through facial expressions. For that reason, we try to assess the degree of symptoms severity in patients with mental disorders based on their social behavior and cognitive functioning, while they are conducting the weekly interviews with the clinicians. Specifically, we aim to automatically recognize the alterations in psychopathology, which are determined through the Positive and Negative Syndrome Scale (PANSS) [40], using features extracted from the patients’ facial expressions [125].

**Data Collection:** PANSS is one of the most well-established procedures to assess symptoms’ severity. Through the monthly in-person assessments with the patients (Section 4), the clinicians are evaluating three types of symptoms, the positive ones that refer to the excessive occurrence of normal functions, and the negative ones which correspond to the limited occurrence of normal functions, as well as general psychopathology symptoms. Overall, 30 symptoms are rated on a scale from 1–7, resulting in a maximum score of 210 points. Due to their association with facial expressions, 10 PANSS elements were chosen to constitute the ground truth for our model, namely: excitement, hostility (positive items) anxiety, poor impulse, motor retardation, depression, tension (general items), blunted effect, poor rapport, lack of spontaneity, and flow of conversation (negative items).

To ensure the correlation between the recorded interview videos and the PANSS values, annotated by the clinicians, we only used videos that were recorded up to two weeks or closer to the assessments that the patients undergo each month. Thus, at the time that this work was conducted, the number of such videos was 167, collected up to early October 2020, (with a duration of up to 1141 seconds) corresponding to 22 patients (2 with Schizoaffective disorder, 1 with Schizophreniform disorder, 1 with Bipolar II disorder, 12 with Schizophrenia and 6 with Bipolar I disorder). Patient demographics are presented in Table 19.

**Methodology and Data Preprocessing**: In order to predict PANSS values from facial cues detected in the interview videos, we followed a pipeline consisting of the following steps: (i) *detection* of the facial area from the video sessions, (ii) frame-wise *feature extraction*, (iii) *aggregation* of the frame-wise features into a single feature vector for each session and finally (iv) *prediction* of the value of the PANSS from the extracted session-wise feature representations.

In more detail, we first subsample the RGB videos, using a sampling rate of one frame per second. Afterwards, in order to extract the face region for each frame, we utilize a pre-trained Multiple Task Cascaded Neural Network (MTCNN) model [126] to detect the facial area, and then crop each frame accordingly.

For the representation of each frame, we compared two methodologies for feature extraction. In the first case, we utilized the widely used Bag of Visual Words (BOVW) method. In particular, *n* keypoints are detected in each frame using the Speeded Up Robust Features (SURF) algorithm [127]; for each detected keypoint, a feature vector with 64 elements is computed. Afterwards, by using the k-means algorithm, the collection of feature vectors calculated over all videos and all frames is segmented into *k* clusters, with each cluster centroid corresponding to a visual word. The final descriptor computed for each frame is a histogram of *k* values, denoting the number of occurrences for each visual word in the frame. On the other hand, the second approach we evaluated is based on transfer learning. In more detail, we utilize the convolutional front-end of an EfficientNet-B0 [128], that has been pre-trained on ImageNet [129]; the frame-wise representation we obtain at the output of the front-end as a feature vector contains a total of k=1280 elements.

By this point, we have acquired an m×n feature representation for each interview session, where *m* corresponds to the number of extracted frames per session video and *n* to the feature dimensionality (n=k for the BOVW-based feature extraction and n=1280 for the EfficientNet-based methodology). In order to aggregate them into a single feature vector, we repeat the BOVW procedure on the whole set of *n*-dimensional frame descriptors, acquiring thus $k^{\prime }$ centroids and assigning a centroid to each frame via k-means. Afterwards, we again form a histogram of $k^{\prime }$ values for each session video, which contains the relative appearance frequency of each visual word in the interview. Finally, the extracted representations for each interview are classified into the respective PANSS class. To this end, we experiment with three widely-used machine learning models: XGBoost (XGB), Random Forests (RF) and Support Vector Machines (SVM) with a radial basis function (RBF) kernel.

**Experimental Protocol and Results:** Based on the pipeline presented above, for each video, we first extract the facial region from the sampled frames, then obtain a low-level feature representation corresponding to each video frame, and finally fuse the frame-level representations into a high-level feature representation for each video. Thus, the following configurations were evaluated: (i) $BOVW^{2}$ (Bag of Visual Words (BOVW) for both low-level and high-level representations) and (ii) EfficientNet to BOVW (EfficientNet features for the low-level representation and BOVW for the high-level representation). The data were divided into two subsets, with 70% of the videos used for training and the rest for testing, whereas the optimal number of clusters for each PANSS item was determined by grid search. The results are presented in Table 20, using the balanced accuracy and the top-2 accuracy as metrics; next to each PANSS element, we also denote the number of values (classes) the respective element takes in the dataset. In terms of balanced accuracy and the $BOVW^{2}$ configuration, the best results concern predictions of tension, hostility and poor impulse control recording values up to 0.72, while anxiety and poor rapport cannot be successfully estimated by either configuration. Additionally, some PANSS items such as poor impulse control, poor rapport, tension and excitement exceed in terms of the top-2 accuracy barrier of 80%, deviating significantly from the balanced accuracy scores, potentially because of the subjectivity of PANSS questionnaire scoring. Finally, we note that contrary to our assumptions, the features extracted from the pretrained EfficientNet (used in the EfficientNet to BOVW configuration) perform worse than those computed by the SURF algorithm (utilized in the $BOVW^{2}$ configuration).

## 6. Conclusions

This paper presents the e-Prevention system, an innovative integrated system for medical monitoring and support that facilitates effective monitoring and relapse prevention in patients with mental disorders. To achieve this target, advanced modules were developed concerning (a) the long-term continuous recording of biosignals through an unobtrusive smartwatch device, (b) the recording of audio-visual data obtained from the weekly interviews that were conducted between patients and clinicians, and (c) the deployment of the cloud server that supports the automatic uploading and storage of all recorded data along with all peripheral modules concerning the dedicated applications that were implemented. We experimentally validated the feasibility of detecting and predicting psychopathology and relapses from both physiological signals acquired from the smartwatch and audiovisual data obtained through the recorded interviews between the patients and the clinicians.

In detail, both traditional and nonlinear features derived from the physiological signals were shown to statistically significantly differ between a group of patients and controls. Based on the above features, we developed and experimentally evaluated a number of state-of-the-art autoencoders for the task of detecting relapses from physiological signals, achieving promising results in personalized experiments. Regarding the audiovisual interviews, convolutional autoencoder architectures using the spontaneous speech of the patients were utilized to detect and predict relapses in both personalized and global settings, with CVAEs in particular successfully scaling in a patient-independent setup. Furthermore, facial cues of the patients, extracted from the interview videos, were found to correlate with psychopathological scales indicative of the patients’ state. Finally, in the case of speech signals, experiments indicate that fusion between speech and biosignals can further bolster the results. Overall, the above results constitute a significant step towards the goal of successful prediction of possible relapses, eventually leading to the improvement of the life quality of mental patients through their prevention.

## Figures and Tables

**Table 1:** Data collected from the smartwatch sensors.

Sensor	Data	Measurement Unit	Frequency Sampling
Accelerometer	Linear Acceleration (3-axis)	m/s${}^{2}$	20 Hz
Gyroscope	Angular Acceleration (3-axis)	degrees/s${}^{2}$	20 Hz
Heart Rate	Heart Rate Variability RR-Intervals	beats/min seconds	5 Hz
Step Counter	Steps and Total distance	steps/min	Total number per minute
Sleep	Sleeping schedule	min	

**Table 2:** Demographics information of controls and patients at the time of recruitment, illness information and amount of recorded data for each group during wakefulness and sleep (up to April 2022).

	Controls	Patients
**Demographics**		
Male/Female	12/11	26/12
Age (years)	27.8 ± 3.9	30.55 ± 7.28
Education (years)	16.9 ± 1.8	13.36 ± 2.18
Illness dur. (years)	-	7.34 ± 6.41
**Recorded Data**		
# Months Recorded	2.81 ± 1.03	16.39 ± 7.36
# 5 min mov (awake)	15,746 ± 4837	37,778 ± 23,429
# 5 min HRV (awake)	12,909 ± 3589	34,548 ± 22,873
# 5 min mov (sleep)	7670 ± 2606	27,377 ± 16,722
# 5 h HRV (sleep)	6924 ± 2331	26,224 ± 16,293

**Table 3:** Demographics information of controls and patients at the time of recruitment, illness information, and amount of recorded data for each group during wakefulness and sleep. There were no significant differences between the amounts of recorded data (tested with the Student’s *t*-test and Shapiro–Wilk for normality).

	Controls	Patients
**Demographics**		
Male/Female	12/11	16/8
Age (years)	27.8 ± 3.9	30.8 ± 6.56
Education (years)	16.9 ± 1.8	13.88 ± 2.27
Illness dur. (years)	-	7.42 ± 5.63
BMI	22.9 ± 3.2	28.25 ± 5.13
PANSS (overall)	-	57.08 ± 14.10
**Recorded Data**		
# Days Recorded	84.3 ± 30.9	68.5 ± 41.7
# 5 min mov (awake)	15,746 ± 4837	13,210 ± 6908
# 5 min HRV (awake)	12,909 ± 3589	12,221 ± 6656
# 5 min mov (sleep)	7670 ± 2606	8865 ± 4767
# 5 h HRV (sleep)	6924 ± 2331	8578 ± 4741

**Table 4:** Statistical difference analysis using Mann-Whitney U-tests with BH correction in each state (wakefulness, sleeping. Bold values denote significance at the 95% confidence levels. For each group the median and the IQR (in parenthesis) are shown for each feature.

		Wakefulness			Sleeping		
	**Feature**	**Controls**	**Patients**	***p*** **Value**	**Controls**	**Patients**	***p*** **Value**
acc	STE mean	6.517 (1.058)	5.832 (2.902)	0.15	0.708 (0.228)	0.406 (0.129)	<**0.001**
	STE std	**8.065 (2.174)**	**6.694 (2.147)**	**0.02**	2.199 (1.375)	1.057 (0.393)	<**0.001**
gyr	STE mean	4045 (1080)	3431 (2313)	0.14	372 (177.542)	185.166 (97.117)	<**0.001**
	STE std	**5572** (**2125**)	**4110** (**2728**)	**0.05**	1324 (1032)	578 (235.108)	<**0.001**
hrv	sampen mean	**1.446** **(0.217)**	**1.260** **(0.281)**	**0.03**	1.435 (0.180)	1.505 (0.169)	0.14
	sampen std	**0.407** **(0.052)**	**0.452** **(0.059)**	**0.03**	0.370 (0.063)	0.376 (0.117)	0.61
	higuchi mean	1.974 (0.010)	1.966 (0.017)	0.07	1.875 (0.067)	1.915 (0.086)	0.18
	higuchi std	0.040 (0.007)	0.043 (0.016)	0.17	0.089 (0.020)	0.079 (0.024)	0.41
	sd1 mean	214.040 (20.128)	194.322 (33.829)	0.08	78.814 (29.462)	72.437 (16.211)	0.61
	sd1 std	**56.058 (7.166)**	**63.894 (9.414)**	**0.02**	60.625 (21.202)	55.604 (14.806)	0.45
	sd2 mean	237.053 (23.944)	219.853 (41.005)	0.14	112.232 (26.737)	104.269 (29.907)	0.32
	sd2 std	**58.511 (6.954)**	**67.642 (13.689)**	**0.01**	63.169 (13.386)	61.827 (8.968)	0.94
	lf/hf mean	0.449 (0.001)	0.449 (0.001)	0.48	0.445 (0.002)	0.445 (0.005)	0.93
	lf/hf std	**0.066 (0.001)**	**0.067 (0.001)**	**0.02**	**0.066 (0.001)**	**0.067 (0.001)**	**<0.001**
	lf mean	30.857 (0.062)	30.858 (0.067)	0.43	30.652 (0.107)	30.644 (0.236)	0.84
	lf std	**3.109 (0.018)**	**3.130 (0.031)**	**0.02**	**3.134 (0.047)**	**3.192 (0.043)**	**<0.001**
	hf mean	69.143 (0.062)	69.142 (0.067)	0.43	69.348 (0.107)	69.356 (0.236)	0.84
	hf std	**3.109 (0.018)**	**3.130 (0.031)**	**0.02**	**3.134 (0.047)**	**3.192 (0.043)**	**<0.001**
	mfd mean mean	1.696 (0.035)	1.655 (0.055)	0.05	1.529 (0.046)	1.516 (0.069)	0.26
	mfd mean std	**0.093 (0.014)**	**0.108 (0.021)**	**0.01**	0.085 (0.023)	0.086 (0.013)	0.93
	mfd std mean	0.035 (0.002)	0.034 (0.004)	0.08	0.027 (0.004)	0.027 (0.004)	0.97
	mfd std std	0.014 (0.002)	0.015 (0.001)	0.34	0.015 (0.003)	0.015 (0.002)	0.93
	mfd mean	1.614 (0.036)	1.590 (0.047)	0.08	1.506 (0.060)	1.502 (0.065)	0.94
	mfd std	**0.071 (0.010)**	**0.078 (0.010)**	**0.03**	0.070 (0.014)	0.067 (0.011)	0.93
	mfd max mean	1.734 (0.032)	1.692 (0.053)	0.06	1.565 (0.047)	1.557 (0.066)	0.36
	mfd max std	**0.094 (0.016)**	**0.110 (0.020)**	**0.02**	0.090 (0.022)	0.088 (0.012)	0.80
	mfd min mean	1.612 (0.035)	1.582 (0.051)	0.05	1.481 (0.040)	1.465 (0.066)	0.41
	mfd min std	**0.076 (0.010)**	**0.089 (0.020)**	**0.01**	0.075 (0.014)	0.077 (0.013)	0.97
walk	steps mean	**7054 (2358)**	**3960 (2928)**	**0.01**	-	-	-
	steps std	**3513** (**1505**)	**2755** (**756**)	**0.05**	-	-	-
sleep	ratio mean	-	-	-	**0.579 (0.107)**	**0.886 (0.471)**	**<0.001**
	ratio std	-	-	-	**0.240 (0.149)**	**0.389 (0.304)**	**0.01**

**Table 5:** Demographics information.

Male/Female	6/4
Age (years)	30.60±7.31
Education (years)	13.8±1.99
Illness dur. (years)	7.3±7.06

**Table 6:** Results for PR-AUC (personalized scheme). ${}^{1}$

Patients	FNN	CNN	Transformer	GRU	Random
#1	0.94	0.95	0.97	0.91	0.91
#2	0.05	0.04	0.02	0.03	0.03
#3	0.54	0.46	0.43	0.44	0.53
#4	0.26	0.34	0.18	0.19	0.18
#5	0.63	0.57	0.60	0.61	0.63
#6	0.70	0.72	0.63	0.67	0.68
#7	0.82	0.86	0.87	0.85	0.86
#8	0.83	0.87	0.65	0.81	0.85
#9	0.79	0.80	0.45	0.75	0.68
#10	0.97	0.95	0.94	0.97	0.97
**Median**	0.75	0.76	0.61	0.71	0.68

^1^ The best results for each patient are shown in bold.

**Table 7:** Results for ROC-AUC (personalized scheme). ${}^{1}$

Patients	FNN	CNN	Transformer	GRU	Random
#1	0.94	0.96	0.97	0.93	0.91
#2	0.49	0.40	0.22	0.36	0.28
#3	0.57	0.53	0.49	0.49	0.52
#4	0.39	0.39	0.35	0.29	0.22
#5	0.44	0.28	0.45	0.40	0.42
#6	0.49	0.49	0.39	0.42	0.48
#7	0.56	0.69	0.69	0.64	0.62
#8	0.72	0.78	0.64	0.60	0.72
#9	0.78	0.75	0.28	0.58	0.42
#10	0.91	0.88	0.81	0.94	0.91
**Median**	0.57	0.61	0.47	0.54	0.50

^1^ The best results for each patient are shown in bold.

**Table 8:** Results for PR and ROC AUC (Global Scheme (Global) and Global scheme evaluated individually (Median)). ${}^{1}$

	FNN	CNN	Transformer	GRU	Random
PR AUC					
**Median**	0.77	0.71	0.76	0.73	0.68
**Global**	0.48	0.49	0.47	0.52	0.50
ROC AUC					
**Median**	0.62	0.58	0.52	0.57	0.50
**Global**	0.47	0.51	0.45	0.53	0.50

^1^ The best results for each architecture are shown in bold.

**Table 9:** Ablation study on the feature modalities (heart rate, accelerometer and gyroscope, or both) used, for the CNN-AE architecture.

Acc./Gyr.	Heart Rate	PR-AUC	ROC-AUC
✓	✓	**0.76**	0.61
✗	✓	0.71	**0.62**
✓	✗	0.73	0.52

**Table 10:** Demographics information at the time of recruitment, illness information, and amount of recorded and analyzed data utterances. ${}^{1}$

Demographics	
Male/Female	10/6
Age (years)	28.7 ± 7.6
Education (years)	13.0 ± 1.7
Illness dur. (years)	7.8 ± 6.8
**Recorded Data**	
Num. of Interviews (total)	474
Num. of Interviews (mean ± std)	29.6 ± 8.1
Diarized speech duration (in s)	38,066
Diarized speech duration (in s, mean ± std)	2379 ± 1444
Num. of Utterances (total)	14,562
Num. of Utterances (mean ± std)	910 ± 485
Num. of Utterances (clean, mean ± std)	766 ± 394
Num. of Utterances (pre-relapse, mean ± std)	119 ± 126
Num. of Utterances (relapse, mean ± std)	169 ± 162

^1^ Utterance statistics for pre-relapsing, or relapsing periods calculated only for those patients who have experienced relapses.

**Table 11:** Per-patient median anomaly scores for the discrimination between sessions that correspond to stable (C), pre-relapsing (P) or relapsing (R) condition, for both CVAE and CAE personalized models. ${}^{1}$

Pat.	CAE			CVAE (MSE)			CVAE (KL)		
ID	C	P	R	C	P	R	C	P	R
#1	0.292 ± 0.035	**0.321** ± 0.067	**0.313** ± 0.065	0.520 ± 0.040	**0.544** ± 0.073	**0.535** ± 0.088	18.60 ± 2.26	**21.67** ± 3.81	16.77 ± 3.89
#2	0.452 ± 0.065	**0.577** ± 0.000	**0.464** ± 0.064	0.703 ± 0.074	**0.880** ± 0.000	0.662 ± 0.063	12.44 ± 3.23	**18.17** ± 0.00	10.10 ± 1.58
#3	0.417 ± 0.068	**0.456** ± 0.065	**0.504** ± 0.061	0.689 ± 0.101	**0.769** ± 0.068	**0.879** ± 0.043	18.49 ± 3.62	**22.30** ± 3.20	**26.45** ± 8.26
#4	0.273 ± 0.034	**0.359** ± 0.070	**0.295** ± 0.027	0.606 ± 0.065	**0.729** ± 0.102	**0.622** ± 0.046	14.38 ± 1.51	**24.53** ± 12.60	**21.46** ± 2.10
#5	0.308 ± 0.029	**0.389** ± 0.010	0.286 ± 0.000	0.595 ± 0.044	**0.706** ± 0.019	0.569 ± 0.000	15.03 ± 1.65	**27.98** ± 2.92	13.71 ± 0.00
#6	0.649 ± 0.104	0.646 ± 0.130	0.599 ± 0.146	0.887 ± 0.091	**0.896** ± 0.190	0.835 ± 0.181	12.43 ± 3.03	**16.23** ± 7.57	**17.48** ± 4.35
#7	0.320 ± 0.018	**0.386** ± 0.048	**0.380** ± 0.000	0.573 ± 0.033	**0.669** ± 0.035	**0.632** ± 0.000	14.03 ± 1.95	**20.17** ± 5.85	**14.45** ± 0.00
#8	0.520 ± 0.051	**0.573** ± 0.000	**0.665** ± 0.000	0.776 ± 0.079	**0.815** ± 0.000	**0.981** ± 0.000	13.32 ± 2.28	**16.12** ± 0.00	**20.79** ± 0.00

^1^ Anomaly scores, corresponding to either pre-relapsing or relapsing states, that follow the desired trend (higher number than stable condition) are shown in bold.

**Table 12:** Average of the per-patient ROC AUC scores for the discrimination between sessions that correspond to stable, or anomalous (pre-relapsing or relapsing) condition, for both CVAE and CAE personalized models.

Pooling	CAE	CVAE	
Function		MSE	KL
AP	**0.681** ± 0.051	**0.669** ± 0.048	0.644 ± 0.052
MP	0.624 ± 0.056	0.595 ± 0.053	0.631 ± 0.054
NP	0.644 ± 0.056	0.640 ± 0.049	**0.659** ± 0.053

**Table 13:** Per-patient ROC AUC scores for the discrimination between sessions that correspond to stable, or anomalous, condition, for both CVAE and CAE personalized models.

Patient	CAE	CVAE	
ID		MSE	KL
#1	0.549 ± 0.140	**0.537** ± 0.111	0.549 ± 0.115
#2	**0.465** ± 0.133	0.433 ± 0.127	0.405 ± 0.127
#3	**0.718** ± 0.171	0.720 ± 0.162	0.662 ± 0.179
#4	0.665 ± 0.064	0.650 ± 0.092	**0.742** ± 0.087
#5	0.780 ± 0.063	**0.800** ± 0.063	0.786 ± 0.068
#6	0.489 ± 0.148	0.512 ± 0.115	**0.656** ± 0.142
#7	0.883 ± 0.082	**0.929** ± 0.090	0.701 ± 0.149
#8	0.790 ± 0.245	0.770 ± 0.237	**0.770** ± 0.256
Average	0.681 ± 0.051	0.669 ± 0.048	**0.659** ± 0.053

**Table 14:** Median anomaly scores for the discrimination between sessions that correspond to stable (C), pre-relapsing (P) or relapsing (R) condition, for both CVAE and CAE global models.

Pers.	Pool.	CAE			CVAE (MSE)			CVAE (KL)		
Norm	Func.	C	P	R	C	P	R	C	P	R
✗	AP	0.166 ± 0.026	0.165 ± 0.038	0.152 ± 0.030	0.483 ± 0.058	0.472 ± 0.086	0.462 ± 0.071	13.07 ± 1.41	**13.47** ± 1.44	12.33 ± 1.28
✗	MP	0.280 ± 0.055	**0.292** ± 0.082	0.255 ± 0.064	0.774 ± 0.120	**0.804** ± 0.176	0.722 ± 0.139	21.58 ± 3.68	**24.79** ± 4.94	**21.78** ± 4.22
✗	NP	0.213 ± 0.036	**0.223** ± 0.053	0.196 ± 0.047	0.591 ± 0.076	**0.610** ± 0.121	0.556 ± 0.103	15.98 ± 2.27	**17.74** ± 2.71	15.65 ± 2.37
✓	AP	0.189 ± 0.030	**0.201** ± 0.046	0.189 ± 0.039	0.520 ± 0.065	**0.581** ± 0.086	**0.537** ± 0.107	12.66 ± 1.03	**14.21** ± 0.99	**12.84** ± 1.52
✓	MP	0.341 ± 0.074	**0.388** ± 0.142	0.339 ± 0.094	0.905 ± 0.176	**1.197** ± 0.420	**1.139** ± 0.411	22.31 ± 4.27	**31.64** ± 9.21	**28.50** ± 7.43
✓	NP	0.252 ± 0.047	**0.283** ± 0.089	0.249 ± 0.066	0.672 ± 0.107	**0.848** ± 0.238	**0.772** ± 0.221	15.86 ± 2.24	**21.65** ± 2.71	**18.85** ± 4.16

**Table 15:** ROC AUC scores for the discrimination between sessions that correspond to stable, or anomalous, condition, for both CVAE and CAE global models, depending on the normalization protocol and pooling function used.

Pers.	Pool.	CAE	CVAE	
Norm	Func.		MSE	KL
✗	AP	0.480 ± 0.016	0.474 ± 0.017	**0.507** ± 0.019
✗	MP	0.525 ± 0.024	0.519 ± 0.021	**0.576** ± 0.026
✗	NP	0.507 ± 0.022	0.501 ± 0.018	**0.558** ± 0.023
✓	AP	0.525 ± 0.025	0.583 ± 0.034	**0.645** ± 0.029
✓	MP	0.531 ± 0.021	0.633 ± 0.036	**0.694** ± 0.032
✓	NP	0.525 ± 0.021	0.625 ± 0.036	**0.698** ± 0.030

**Table 16:** Average ROC-AUC scores for the discrimination between stable and anomalous (pre-relapsing or relapsing) patients’ condition for the HRV autoencoder, depending on the architecture, normalization scheme and anomaly score.

Architecture	Pers. Norm	EMD	KL
CNN-AE	✗	0.506 ± 0.051	-
CNN-AE	✓	0.589 ± 0.086	-
1D-CVAE	✗	0.492 ± 0.051	0.471 ± 0.042
1D-CVAE	✓	0.438 ± 0.128	**0.666** ± 0.073

**Table 17:** Average ROC-AUC scores for the discrimination between stable and anomalous (pre-relapsing or relapsing) patients’ condition, depending on the modalities and fusion.

Audio	HRV	Fusion	ROC-AUC
✓	✗	-	0.764 ± 0.045
✗	✓	-	0.666 ± 0.073
✓	✓	Mult.	0.773 ± 0.041
✓	✓	Add.	**0.779** ± 0.038

**Table 18:** Average ROC-AUC scores for the discrimination between stable and anomalous (pre-relapsing or relapsing) patients’ condition, depending on the temporal pooling per modality.

Audio	HRV Pooling		
Pooling	AP	MP	NP
AP	0.729 ± 0.038	0.602 ± 0.064	0.629 ± 0.057
MP	0.764 ± 0.036	0.707 ± 0.036	0.723 ± 0.037
NP	**0.779** ± 0.038	0.696 ± 0.040	0.718 ± 0.038

**Table 19:** Demographics information.

Male/Female	16/6
Age (years)	30.36±7.51
Education (years)	12.95±2.03
Illness dur. (years)	8.32±6.98

**Table 20:** Classification results for $BOVW^{2}$ (left) and EfficientNet to BOVW (right) configurations based on balanced accuracy (left) and top-2 accuracy (right) metrics. The number of respective classes is shown next to PANSS items. ${}^{1}$

PANSS Items	${\mathrm{BOVW}}^{2}$						EfficientNet to BOVW					
	RF		XGB		SVM		RF		XGB		SVM	
Depression (6c)	0.49	0.82	**0.6**	0.85	0.30	0.68	0.47	0.64	0.53	0.64	0.30	0.75
Anxiety (5c)	**0.44**	0.65	0.37	0.69	0.33	0.84	0.26	0.77	0.36	0.77	0.29	0.85
Tension (4c)	**0.70**	0.89	0.68	0.85	0.56	0.62	0.54	0.77	0.61	0.85	0.46	0.81
Poor Rapport (4c)	0.44	0.86	0.49	0.90	0.34	0.68	0.41	0.86	0.55	0.82	0.34	0.57
Poor Impulse Control (3c)	**0.72**	1	0.71	0.79	0.39	1	0.43	0.96	0.66	0.82	0.37	0.96
Motor Retardation (4c)	0.40	0.75	**0.60**	0.82	0.36	0.75	0.41	0.82	0.40	0.79	0.32	0.79
Excitement (4c)	0.58	0.78	**0.61**	0.82	0.42	0.78	0.49	0.85	0.47	0.85	0.41	0.67
Hostility (3c)	**0.72**	0.96	0.68	0.93	0.42	0.78	0.42	1	0.60	0.89	0.49	0.79
Blunted Affect (5c)	**0.58**	0.68	0.55	0.64	0.34	0.64	0.34	0.64	0.47	0.68	0.30	0.64
Lack of Spontaneity(5c)	0.49	0.64	**0.65**	0.79	0.40	0.75	0.49	0.64	0.26	0.68	0.43	0.75

^1^ Best results, regarding the balanced accuracy metric, are shown in bold for each configuration and classification method.

## Data Availability

Restrictions apply to the availability of the data used in this study, due to privacy issues, the nature of the mental state of the patients and the fact that the project is still in effect.
